# Mismatch repair protein MLH1 controls testis development by regulating the Hippo-YAP signaling pathway

**DOI:** 10.1093/nar/gkag883

**Published:** 2026-09-09

**Authors:** Xueying Li, Jiajun Yang, Guo-Min Li

**Affiliations:** Beijing Key Laboratory of Tumor Resistance Mechanism and Clinical Translation, Chinese Institutes for Medical Research, Beijing 100069, China; Chinese Institute for Cancer Research, Chinese Institutes for Medical Research, Beijing 100069, China; Beijing Key Laboratory of Tumor Resistance Mechanism and Clinical Translation, Chinese Institutes for Medical Research, Beijing 100069, China; Chinese Institute for Cancer Research, Chinese Institutes for Medical Research, Beijing 100069, China; School of Basic Medical Sciences, Capital Medical University, Beijing 100069, China; Beijing Key Laboratory of Tumor Resistance Mechanism and Clinical Translation, Chinese Institutes for Medical Research, Beijing 100069, China; Chinese Institute for Cancer Research, Chinese Institutes for Medical Research, Beijing 100069, China; Beijing Chao-Yang Hospital, Capital Medical University, Beijing 100020, China; School of Basic Medical Sciences, Capital Medical University, Beijing 100069, China

## Abstract

DNA mismatch repair (MMR) maintains genomic stability, and defects in MMR genes such as *MLH1* and *MSH2* predispose to cancer. Unlike other MMR components, MLH1 has unexplained roles in development, as *Mlh1*-deficient male mice exhibit severe testicular hypoplasia and sterility. Here, we uncover that MLH1 regulates testis development through the Hippo-Yes-associated protein (YAP) pathway. MLH1 directly binds YAP via its C-terminal domain and the WW domains of YAP, competitively inhibiting LATS1-mediated YAP phosphorylation. This interaction stabilizes YAP by suppressing ubiquitination and promotes its nuclear translocation dependent on MLH1’s nuclear localization signal. Additionally, MLH1 facilitates YAP–TEAD complex formation, enabling expression of testicular development genes, including *Wt1, Sox9*, and *Ctgf*. These functions are independent of the MMR activity of MLH1. *Mlh1*-deficient mice show elevated YAP phosphorylation, reduced target gene expression, and impaired proliferation in developing testes. Pharmacological inhibition of the Hippo pathway kinases MST1/2 partially rescues testis hypoplasia in *Mlh1*^−/−^ mice. These findings establish MLH1 as a Hippo pathway regulator and resolve its long-standing role in male gonad development.

## Graphical Abstract

**Graphical Abstract:**
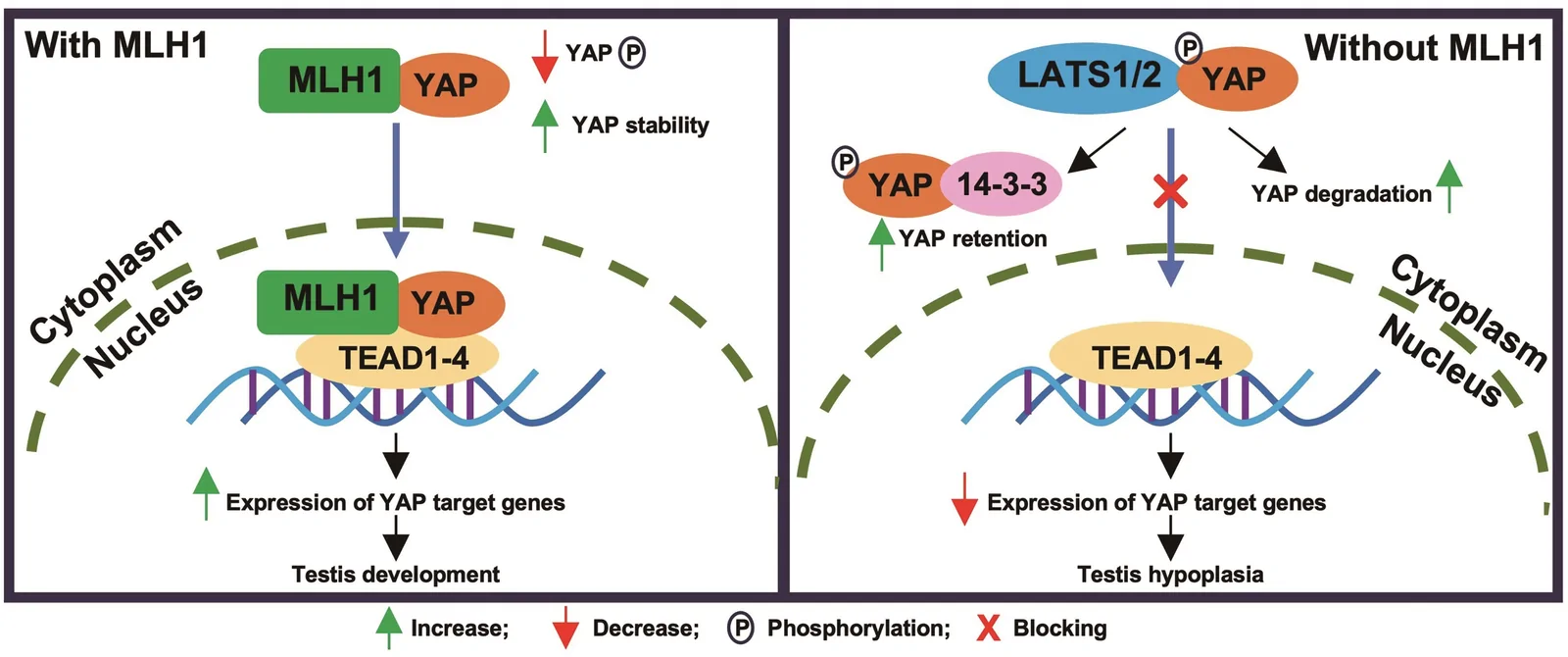

## Introduction

DNA mismatch repair (MMR) is essential for maintaining genomic stability by correcting replication errors [1–4]. Mutations in MMR genes, including *MLH1, MSH2*, and *MSH6*, are well-known drivers of cancers displaying microsatellite instability (MSI) [5–7]. However, unlike other core MMR components, which are exclusively involved in MMR, MLH1 has been implicated in biological processes beyond canonical MMR, such as DNA recombination, immune signaling, and meiosis [8–13]. Surprisingly, *Mlh1*-deficient male mice are sterile and exhibit severe testicular hypoplasia [12, 13]. This phenotype cannot be fully explained by defective meiosis alone, suggesting a separate and critical role for MLH1 in testis development. The molecular mechanism underlying the disturbed testicular development in *Mlh1*-deficient animals, however, remains unknown.

The Hippo-Yes-associated protein (YAP) signaling pathway is a key regulator that controls organ size and tissue homeostasis by modulating cell proliferation and apoptosis [14–18]. In mammals, this pathway is comprised of a core kinase cascade, where mammalian sterile 20-like kinase 1/2 (MST1/2) phosphorylates and activates large tumor suppressor kinase 1/2 (LATS1/2) [19–24]. The activated LATS1/2 in turn phosphorylates and inactivates the transcriptional coactivator YAP and its paralog TAZ. YAP phosphorylation promotes its cytoplasmic retention and subsequent degradation [25, 26]. Unphosphorylated YAP goes into the nucleus, where YAP interacts with the TEAD (TEA/ATTS domain) family transcription factors (TEAD1–4) [27, 28] to promote the expression of a number of genes, including those involved in organ development. Overexpression of YAP or depletion of its upstream tumor suppressors causes uncontrolled growth in many organs, suggesting that YAP promotes organ growth/development [16]. Consistent with this notion, conditional knockout of YAP/TAZ leads to an increase in apoptotic cells and a decrease in testis size [29]; loss of the YAP function leads to severe testis hypoplasia, abnormal spermatozoa, and complete infertility in male mice [30], mirroring the phenotype observed in *Mlh1*-deficient mice. This striking similarity prompted us to investigate a potential mechanistic link between MLH1 and YAP. We hypothesize that MLH1 regulates testis development by modulating the Hippo-YAP pathway.

Here, we demonstrate that MLH1 directly interacts with YAP, shielding it from LATS1-mediated phosphorylation and promoting its nuclear translocation and transcriptional activity. Loss of MLH1 results in excessive YAP degradation, disrupting the expression of genes essential for testicular development and leading to severe testis hypoplasia. These findings reveal a novel, non-canonical function for MLH1 and resolve the long-standing question surrounding its indispensable role in male gonad development.

## Materials and methods

### Mouse strains and genotyping

All animal procedures were reviewed and approved by the Animal Care and Use Committees of the Chinese Institutes for Medical Research, Beijing (AEEl-2025–925). The *Mlh1*-deficient mouse model was established by crossing heterozygote B6.129-*Mlh1^tm1Rak^* (*Mlh1*^+/−^), originally provided by X. William Yang (University of California, Los Angeles), to generate *Mlh1*^−/−^ offspring. An independent *Mlh1*-KO strain (Stock No. NM-KO-200596) was obtained from Shanghai Model Organisms Center, Inc. Wild-type and *Msh2*-KO C57BL/6 mice were purchased from the Jackson Laboratory. All experimental mice were housed under a specific pathogen-free environment with 12 h–12 h light–dark cycles and were fed with standard chow and water.

Genotypes of knockout mice and wild-type (WT) littermates were determined by polymerase chain reaction(PCR) analysis of genomic DNA extracted from tail biopsies. For B6.129-*Mlh1^tm1Rak^* mice, we used forward primer TGTCAATAGGCTGCCCTAGG and reverse primers TGGAAGGATTGGAGCTACGG (for *Mlh1^−/−^* mice) and TTTTCAGTGCAGCCTATGCTC (for WT mice) to obtain PCR products with 500- and 350-bp in length for *Mlh1^−/−^* and WT mice, respectively. For *Mlh1*-KO strain (Stock No. NM-KO-200596), we used forward primers TGCTCTGTCCTTGCCACTTACG (for *Mlh1*^−/−^ mice) and TAGATCAAAGGCTTGAACACTTCCC (for WT mice) and reverse primer AAGCCCTCCTGAATGACCAATA to obtain PCR products with 477- and 302-bp in length for *Mlh1*^−/−^ and WT mice, respectively. For *Msh2* mice, the genotyping analysis used forward primer TGTGCTGGCTCACTTAGACG and reverse primers ATATTGCTGAAGAGCTTGGCGGC (*Msh2^−/−^* mice) and AAAGTGCACGTCATTTGGA (for WT mice). The PCR products are 181- and 600-bp in length for WT and *Msh2^−/−^* mice, respectively. The cycling conditions were 3 min at 94°C, 35 cycles of 1 min at 94°C, 2 min at 65°C, and 1 min at 72°C, 3 min at 72°C, followed by holding at 4°C.

### Treatment with the MST1/2 inhibitor XMU-MP-1

The MST1/2 kinase inhibitor XMU-MP-1 was purchased from MedChemExpress (HY-100526). For *in vivo* administration, XMU-MP-1 was dissolved in a vehicle solution of 0.1% citric acid in water with 20% Kolliphor HS 15. Eighteen-day-old male WT and *Mlh1^−/-^* mice were randomly assigned to treatment or control groups. Mice in the treatment group received a daily intraperitoneal (I.P.) injection of XMU-MP-1 (1 mg/kg) for 20 consecutive days. Control mice received an equivalent volume of the vehicle solution alone. At the end of the treatment period, mice were euthanized, and testes were collected for subsequent analysis.

### Cell lines and cell culture

Several human (HeLa, U2OS, HCT116, HEK293T, and HEK293) and mouse (TM4 and 15P-1) cell lines were used in this study. HCT116 and U2OS were grown in McCoy’s 5A medium with 10% fetal bovine serum (FBS). HeLa, HEK293T, HEK293, and 15P-1 were cultured in Dulbecco’s modified Eagle’s medium (DMEM) supplemented with 10% FBS. TM4 was grown in a 1:1 mixture of Ham’s F12 medium and DMEM with 1.2 g/l sodium bicarbonate, 15 mM HEPES supplemented with 2.5% FBS and 5% horse serum. 15P-1 cells were maintained at 32°C in a 5% CO_2_ incubator, while all other lines (HeLa, U2OS, HCT116, HEK293T, HEK293, and TM4) were maintained at 37°C in a 5% CO_2_ incubator. Knockout cell lines were generated using CRISPR–Cas9 technologies with sgRNA sequences, as described (Supplementary Table S1) [9, 31]. HeLa-*MLH1^−/−^*-*MLH1*, U2OS-*MLH1^−/−^*-*MLH1*, and HCT116-*MLH1* cell lines were generated by transfecting lentivirus carrying *MLH1* and maintained with 10 µg/ml puromycin. The TM4-*Mlh1^−/−^*-*Mlh1* and 15P-1-*Mlh1^−/−^*-*Mlh1* cell lines were generated by transfecting lentivirus carrying *Mlh1* and maintained with 10 µg/ml puromycin. *MLH3*-knocking down cells were generated by the genOFF h-*MLH3* pack purchased from RiBobio.

### Preparation of protein extracts and western blotting

Cells and tissues were lysed using RIPA lysis buffer [40 mM Tris–HCl, pH 7.4, 150 mM NaCl, 1% NP-40, 0.5% sodium deoxycholate, and 0.1% sodium dodecyl sulfate (SDS)] containing a protease inhibitor cocktail and a phosphatase inhibitor cocktail. Protein concentrations were measured using the BCA protein assay kit (cat. no. 23225, Thermo Fisher). Proteins were separated by sodium dodecyl sulfate–polyacrylamide gel electrophoresis (SDS–PAGE), transferred onto a nitrocellulose membrane, and detected using the indicated antibodies (Supplementary Table S1).

### Immunoprecipitation

Immunoprecipitation (IP) assays were performed using standard protocols. Cells or tissue samples were lysed using RIPA buffer supplemented with protease and phosphatase inhibitor cocktails, with or without benzonase (250 U/ml) to degrade nucleic acids. Lysates were incubated with specific antibody for 2 h at 4°C with gentle agitation. Protein G agarose beads were added, and the incubation continued for an additional 2 h. Beads were then washed three times with ice-cold RIPA buffer. Bound proteins were eluted and analyzed alongside input lysates by subsequent Western blot analysis.

### GST pull-down assay

GST pull-down assays were performed to analyze protein interactions *in vitro*. Recombinant His-tagged MutLα protein was pre-cleared with Glutathione-Sepharose 4B beads (GE Healthcare). The pre-cleared protein was then incubated with the beads pre-bound to the relevant GST fusion protein. Following incubation, the beads were washed three times with RIPA buffer. The bound protein complexes were subsequently eluted by boiling in SDS–PAGE loading buffer and analyzed by Western blot.

### *In vitro* kinase assay

An *in vitro* kinase assay was performed to assess LATS1-mediated phosphorylation of YAP. Recombinant GST-YAP was incubated with either active GST-LATS1-kinase or GST protein (negative control) in kinase buffer (50 mM Tris–HCl, pH 7.5, 15 mM MgCl_2_, 20 mM KCl, 2 mM EDTA, 1 mM DTT, and 500 µM ATP) at 30°C for 30 min in the presence or absence of His-MutLα protein. Reactions were terminated by heating at 96°C in SDS loading buffer. Phosphorylation of YAP was subsequently analyzed by western blot.

### RNA extraction and quantitative real-time PCR

Total RNA was extracted from cultured cells or mouse testes using TRIzol reagent (Invitrogen) according to the manufacturer’s protocol. For each sample, 1 μg of RNA was reverse transcribed into cDNA using qScript cDNA SuperMix (Quantabio). Quantitative real-time PCR (qRT-PCR) was performed using PowerUp SYBR Green Master Mix (Thermo Fisher) on a CFX96 real-time system (Bio-Rad). Relative gene expression levels were normalized to *GAPDH*. Forward and reverse primers for each gene are listed in Supplementary Table S1.

### Confocal microscopy and immunofluorescence analysis

For immunofluorescence, cells were seeded onto glass coverslips. The cells were fixed in 4% formaldehyde for 10 min at room temperature, followed by three washes with PBS. Permeabilization was performed using 0.2% Triton X-100 in PBS for 20 min. Cells were then blocked in PBS containing 5% BSA for 1 h at room temperature. Following blocking, cells were incubated with the indicated primary antibodies diluted in blocking buffer overnight at 4°C. After washing, cells were incubated with the appropriate Alexa Fluor 488- or 555-conjugated secondary antibodies (Invitrogen) for 1 h at room temperature. Nuclei were counterstained with DAPI before mounting. Images were acquired using a ZEISS LSM 980 confocal microscope and processed with ZEN Microscopy Software (ZEISS Microscopy Imaging Systems). Fluorescence intensity was quantified using ImageJ software.

### Histological immunostaining

For histological examination, mouse testes were fixed overnight in 4% formaldehyde in PBS, embedded in paraffin, and then sectioned. Sections were stained with hematoxylin and eosin using standard protocols. For immunohistochemistry (IHC), paraffin-embedded sections were deparaffinized in xylene and rehydrated in a graded series of ethanol solutions. Endogenous peroxidase activity was quenched by incubating sections in 0.3% H_2_O_2_ in PBS for 30 min at room temperature, followed by antigen retrieval. After blocking with 5% normal goat serum, sections were incubated with the primary antibody overnight at 4°C. Signal detection was performed using a rabbit-specific HRP/DAB detection IHC kit (Abcam) according to the manufacturer’s instructions. Images were captured using a Zeiss microscope under appropriate magnification.

### TUNEL staining

Terminal deoxynucleotidyl transferase dUTP nick end labeling (TUNEL) staining was performed on tissue sections using the DeadEnd™ Colorimetric TUNEL System (Promega), according to the manufacturer’s instructions. Following the TUNEL reaction, sections were counterstained with hematoxylin solution (Abcam) to visualize nuclei.

### Quantification and statistical analysis

All graphs and statistical analyses were performed using GraphPad Prism software (version 9). Data are presented as individual data points, except for Figs 3D and 4B, Supplementary Figs S1B, S4D, and S6V, which are presented as means ± SD. Significance was determined using a two-tailed unpaired Student’s *t*-test for comparisons between two groups or one-way ANOVA test for comparisons among multiple groups [32]. All experiments were performed with at least three independent biological replicates. A *P*-value below 0.05 was considered statistically significant (ns, no significance; *, *P* < 0.05; **, *P <* 0.01; ***, *P <* 0.001; ^****^, *P <* 0.0001).

## Results

### *Mlh1* ^−/−^ mice exhibit smaller testes and increased YAP phosphorylation

**Figure 1.**
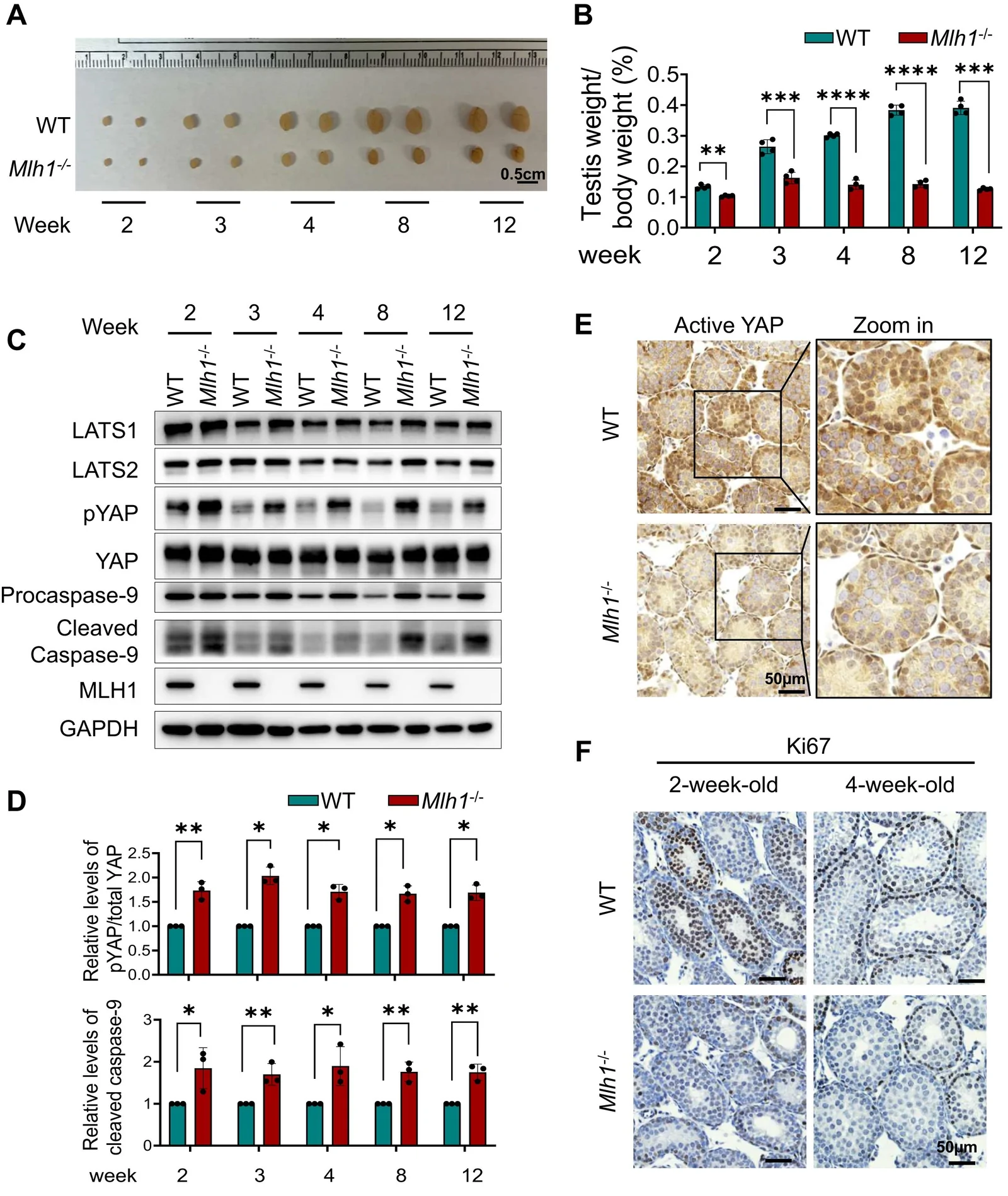
*Mlh1* deficiency causes testicular hypotrophy and promotes YAP hyperphosphorylation.(**A**) Gross morphology of testes from WT and *Mlh1*^−/−^ mice at 2–12 weeks of age. Scale bar, 0.5 cm. (**B**) Relative testis weight (testis/body weight ratio) of WT and *Mlh1*^−/−^ mice (*n* = 4 per genotype at each time point). (**C**) Western blot analysis of testis lysates showing enhanced YAP phosphorylation (pYAP, Ser112) and increased caspase-9 cleavage in *Mlh1^−/−^* mice compared with WT across the indicated ages. Total YAP and procaspase-9 levels are also shown as loading controls. (**D**) Quantification of pYAP/total YAP ratio and cleaved caspase-9/procaspase-9 ratio from (**C**) (*n* = 3 independent experiments). (**E**) IHC staining for active, non-phosphorylated YAP in WT and *Mlh1^−/−^* testes. (**F**) IHC for the proliferation marker Ki-67 in WT and *Mlh1^−/−^* testes. Data are presented as individual data points. *P* values in panels (B) and (D) were determined by a two-tailed unpaired *t*-test with Welch’s correction. *, *P <* 0.05; **, *P <* 0.01; ***, *P <* 0.001; ^****^, *P <* 0.0001.

To investigate the role of MLH1 in testis development, we analyzed *Mlh1*-knockout (*Mlh1^−/−^*) mice. Consistent with previous reports [13], the testes of *Mlh1^−/−^* animals were significantly smaller than those of WT littermates (Fig. 1A and B). This hypoplasia was evident as early as two weeks of age, indicating an early developmental defect, whereas body sizes did not differ between genotypes (Supplementary Fig. S1A and B).

Based on the phenotypic similarity to YAP-deficient testes and our hypothesis that MLH1 regulates the Hippo pathway, we examined YAP activity. In *Mlh1^−/−^* testis tissue, we observed a marked increase in the phosphorylated, inactive form of YAP compared with WT controls (Fig. 1C and D). This was confirmed by IHC, which showed markedly reduced staining of active YAP in *Mlh1^−/−^* testes (Fig. 1E). These results suggest that Mlh1 prevents YAP phosphorylation by LATS1/2.

The developmental defect in *Mlh1^−/−^* testes was further characterized by a severe impairment of cell proliferation. Seminiferous tubules from *Mlh1^−/−^* testes exhibited a marked reduction in the proliferation marker Ki67 at both 2 and 4 weeks of age compared with WT controls (Fig. 1F). This proliferative deficit was accompanied by a concurrent increase in apoptosis at weeks 4 and 8 (Supplementary Fig. S1C) and a loss of intact seminiferous tubule structure (Supplementary Fig. S1D). Consistently, cleaved caspase-9 levels were significantly elevated in *Mlh1^−/−^* testes from 2 to 12 weeks of age (Fig. 1C and D), indicating activation of the intrinsic apoptotic pathway. Notably, these defects were specific to MLH1 loss, as testes from *Msh2^−/−^* mice developed normally and showed no discernable perturbation of the Hippo-YAP pathway (Supplementary Fig. S1E and F).

We next investigated the potential tissue specificity of the MLH1–YAP interaction by analyzing their co-expression. Protein expression profiling across multiple mouse tissues revealed that the testis and liver uniquely exhibited high concurrent levels of both MLH1 and YAP (Supplementary Fig. S1G), suggesting that MLH1-dependent regulation of YAP is most impactful in tissues where both proteins are high-expressed, with testis representing a particularly sensitive context for detecting effects on organ growth. Collectively, these findings, i.e., impaired proliferation, increased apoptosis, and specific co-expression, strongly support the hypothesis that MLH1 modulates testis development through regulating the Hippo-YAP pathway.

### MLH1 interacts with YAP and inhibits YAP phosphorylation

**Figure 2.**
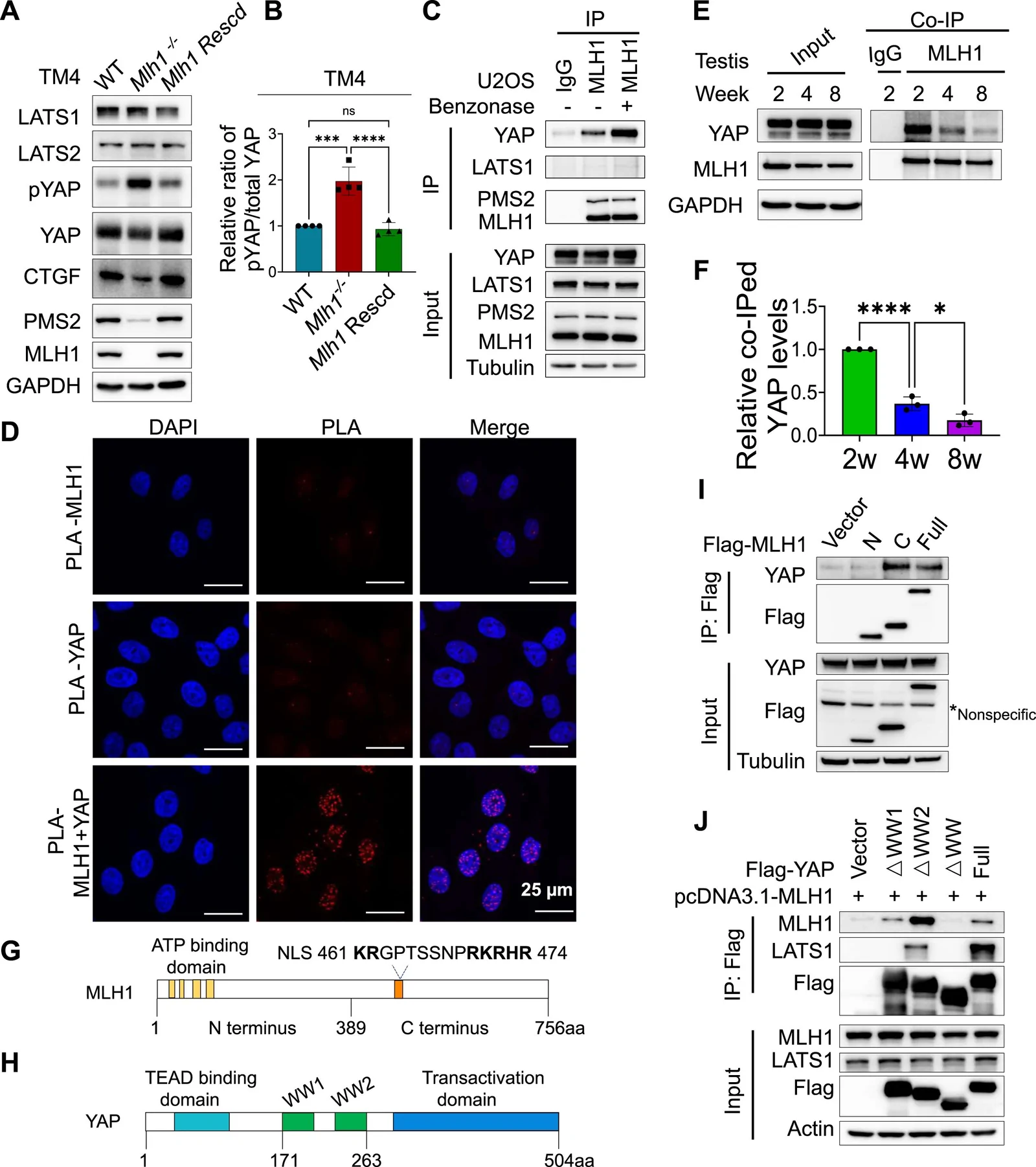
MLH1 directly interacts with YAP both *in vivo* and *in vitro*. (**A**) Western blot analysis of TM4 cell lysates showing increased pYAP, decreased total YAP, and reduced expression of the YAP target gene CTGF in *Mlh1^−/−^* cells compared with WT and *Mlh1*-rescued cells. (**B**) Quantification of pYAP/YAP ratios from panel (A) (*n* = 4 independent biological replicates; mean ± SD). (**C**) Co-immunoprecipitation (Co-IP)–western blot analysis in U2OS cells confirming an interaction between MLH1 and YAP, but not between MLH1 and LATS1. (**D**) Proximity ligation assay (PLA) detecting *in situ* interaction between YAP and MLH1 in HeLa cells. (**E**) Co-IP of endogenous proteins from mouse testis tissue lysates (2, 4, and 8 weeks of age) using an anti-MLH1 antibody confirms the YAP–MLH1 interaction *in vivo*. (**F**) Quantification of co-immunoprecipitated YAP levels from panel (E), normalized to immunoprecipitated MLH1 (*n* = 3 mice per time point). Schematical diagram of the functional domains in MLH1 (**G**) and YAP (**H**). (**I**) Co-IP-Western analysis in HEK293T cells shows that YAP interacts with the C-terminal fragment, but not the N-terminal fragment, of Flag-tagged MLH1. (**J**) Co-IP in HEK293T cells demonstrates that both MLH1 and LATS1 interact with full-length YAP, but not with a YAP mutant lacking its WW domains (ΔWW). Data are representative of at least three independent experiments. Statistical significance in panels (B) and (F) was determined by one-way ANOVA; *, *P <* 0.05; **, *P <* 0.01; ***, *P <* 0.001; ^****^, *P <* 0.0001.

To investigate the mechanism by which MLH1 suppresses YAP phosphorylation, we generated *Mlh1* knockouts (*Mlh1*-KO) in TM4 and 15P-1 mouse Sertoli cells (Fig. 2A and Supplementary Fig. S2A) and also examined multiple *MLH1*-deficient human cell lines, including engineered knockouts in U2OS and HeLa cells as well as the *MLH1*-deficient colorectal line HCT116 (Supplementary Fig. S2B–D). In mouse cells, *Mlh1*-KO destabilized PMS2 but left LATS1/2 expression unchanged (Fig. 2A and Supplementary Fig. S2A), while markedly elevating phosphorylated YAP (pYAP) levels compared with WT or *Mlh1*-rescued controls (Fig. 2A and B and Supplementary Fig. S2A and B), recapitulating observations in *Mlh1^−/−^* testes (Fig. 1C and D). Consistently, all *MLH1*-deficient human cell lines exhibited elevated pYAP, and restoring MLH1 in knockout cells returned pYAP to WT levels (Supplementary Fig. S2B–D). Across all models, MLH1 status did not affect LATS1/2 protein levels (Fig. 2A and Supplementary Fig. S2A–D). Together, these results demonstrate that MLH1 regulates YAP phosphorylation independent of LATS1/2 expression, a finding consistent across species and cell types.

MLH1 functions as an obligate core of distinct MutL complexes by dimerizing with PMS2 (MutLα), PMS1 (MutLβ), or MLH3 (MutLγ) [33–35]. To determine whether regulation of the Hippo-YAP pathway requires a specific MLH1 complex, we assessed YAP phosphorylation following genetic perturbation of each partner protein. In contrast with the pronounced effect of MLH1 loss, depletion of *PMS2, PMS1*, or *MLH3* in HeLa and U2OS cells resulted in minimal changes to YAP phosphorylation (Supplementary Fig. S2E–I), indicating that the core MLH1 subunit is essential for YAP regulation, while its partner proteins (PMS2, PMS1, and MLH3) are functionally redundant for this activity.

To test whether MLH1 regulates YAP phosphorylation through direct interaction, we performed Co-IP assays in U2OS cells. IP of endogenous MLH1 efficiently co-precipitated YAP, but not the upstream kinase LATS1 (Fig. 2C). This interaction was confirmed by reciprocal Co-IP (Supplementary Fig. S3A) and was consistently observed in TM4, 15P-1, and HeLa cells (Supplementary Fig. S3B–D). Treatment with benzonase to eliminate nucleic acids did not disrupt the interaction, confirming that it was not DNA-mediated (Fig. 2C and Supplementary Fig. S3A). The association was further validated by GST pulldown assays with purified proteins (Supplementary Fig. S3E) and PLA demonstrating *in situ* nuclear colocalization (Fig. 2D). This interaction was specific to MLH1, as no binding occurred between YAP and MSH2 (Supplementary Fig. S3F), another key component in MMR. We then examined the interaction in a developmental context. In mouse testis extracts, the MLH1–YAP interaction decreased postnatally (Fig. 2E and F). These data suggest that MLH1–YAP binding is critical during early testis development, leading us to conclude that MLH1 regulates YAP functions via its physical interaction with YAP during early testis development.

To map the interaction domains mediating the MLH1–YAP binding, we assessed the binding capacity of Flag-tagged MLH1 or YAP fragments to full-length YAP or MLH1, respectively (Fig. 2G and H). Co-IP assays revealed that while full-length Flag-MLH1 and Flag-YAP each robustly bound their respective partner (Fig. 2I, lane 4 and Fig. 2J, lane 5), only the C-terminal fragment of MLH1, not the N-terminal fragment, pulled down YAP (Fig. 2I, lane 3). Similarly, deletion of both WW domains in YAP abolished its interaction with MLH1 (Fig. 2J, lane 4). Since the WW domains of YAP are also required for its interaction with the upstream kinases LATS1/2 [25, 36], these data suggest that MLH1 competes with LATS1/2 for YAP binding, thereby inhibiting YAP phosphorylation. Supporting this competitive model, re-expression of full-length MLH1 or its YAP-interacting C-terminal fragment (but not the non-interacting N-terminal fragment) in *MLH1^−/−^* cells was sufficient to suppress YAP phosphorylation (Supplementary Fig. S3G, lanes 5 and 4 versus lane 3).

### The MLH1–YAP interaction extends YAP’s half-life

**Figure 3.**
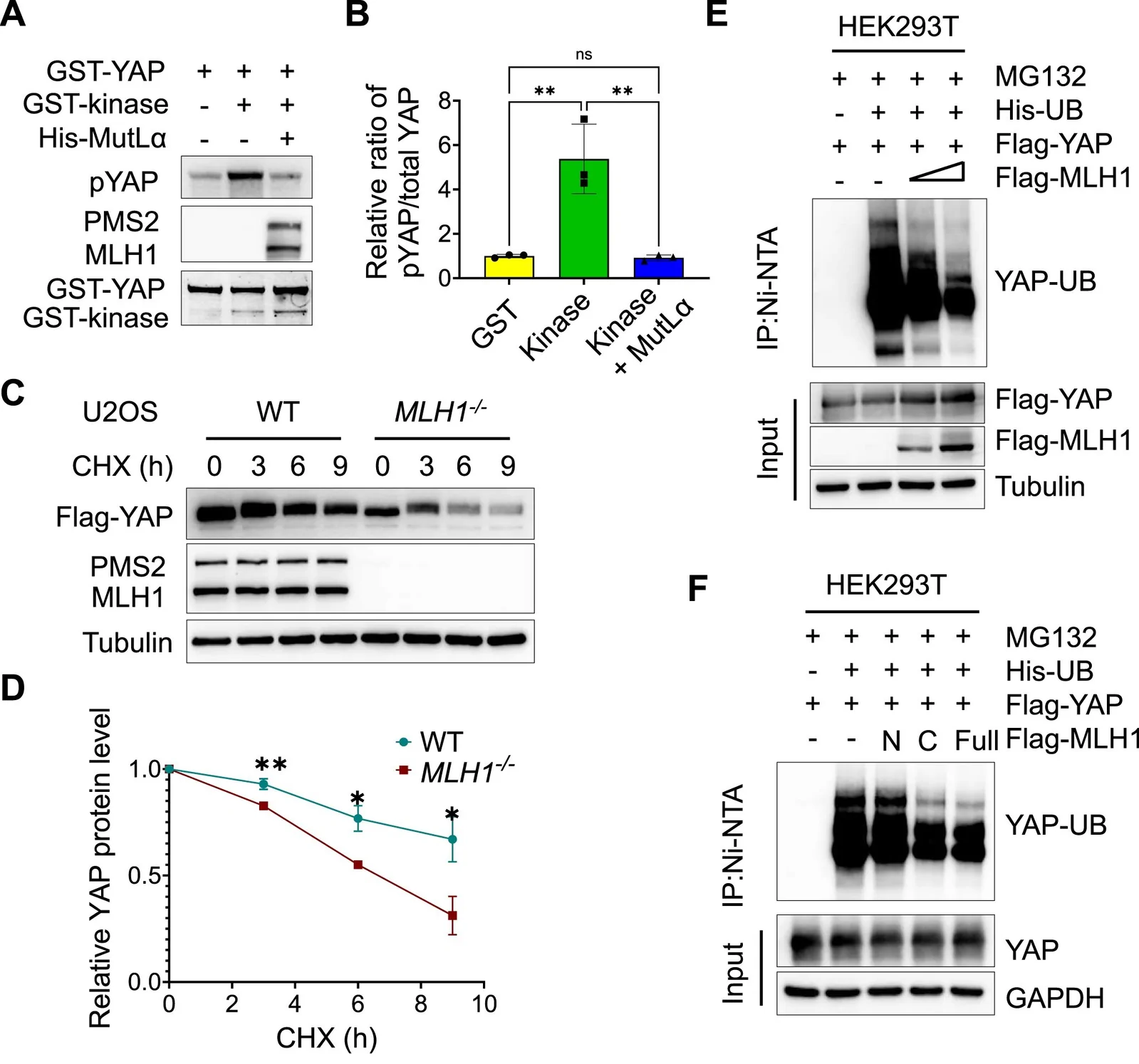
MLH1 binding protects YAP from phosphorylation and ubiquitination. (**A**) *In vitro* kinase assay showing that purified MutLα complex inhibits LATS1-mediated phosphorylation of YAP. (**B**) Quantification of phosphorylated YAP from three independent kinase assays as shown in panel (A). Data are presented as individual data points. (**C**) Assessment of YAP protein stability. WT and *MLH1^−/−^* U2OS cells expressing Flag-YAP were treated with cycloheximide (CHX, 100 μg/mL) for the indicated times. Flag-YAP levels were analyzed by Western blot. (**D**) Quantification of residual Flag-YAP levels from three independent experiments shown in panel (C). Data are presented as mean ± SD. (**E**) *In vivo* ubiquitination assay showing reduced YAP ubiquitination in the presence of MLH1. Flag-YAP and His-ubiquitin were co-expressed in HEK293T cells with increasing amounts of Flag-MLH1. Cells were treated with MG132 (10 μM) for 12 h prior to lysis. Ubiquitinated proteins were isolated by denaturing Ni-NTA pull-down and analyzed by immunoblotting with an anti-YAP antibody. (**F**) *In vivo* ubiquitination assay showing that full-length MLH1 and its C terminal fragment, but not its N terminal fragment, reduce YAP ubiquitination. Statistical significance was determined by one-way ANOVA (B) or a two-tailed unpaired *t*-test with Welch’s correction (D). *, *P <* 0.05; **, *P <* 0.01.

To directly test whether the MLH1–YAP interaction inhibits YAP phosphorylation by LATS1, we conducted *in vitro* kinase assays using purified proteins. As expected, the GST-LATS1 kinase domain robustly phosphorylated GST-YAP (Fig. 3A, lane 2). However, addition of the MutLα complex (MLH1-PMS2) significantly reduced YAP phosphorylation (Fig. 3A and B), indicating that MLH1 binding competitively blocks LATS1-mediated phosphorylation. Consistent with this notion, YAP–LATS1 interaction was enhanced in *MLH1^−/−^* U2OS cells (Supplementary Fig. S4A) and diminished in HEK293T cells overexpressing MLH1 (Supplementary Fig. S4B).

Because YAP phosphorylation triggers its proteasomal degradation [26], we postulated that MLH1 functions to stabilize the YAP protein. CHX chase assays in U2OS cells revealed that YAP had a significantly shorter half-life in *MLH1^−/−^* cells compared with WT controls (Fig. 3C and D), a phenotype recapitulated in HCT116 isogenic lines (Supplementary Fig. S4C and D). Consistent with this, ubiquitination assays showed markedly reduced YAP polyubiquitination in *MLH1*-rescued 293T cells compared with *MLH1*-deficient controls (Fig. 3E, lane 3 versus 2). This effect was dose-dependent, as MLH1 overexpression further suppressed ubiquitination (Fig. 3E, lane 4). Critically, reduced ubiquitination required the MLH1–YAP interaction, as only full-length MLH1 or its YAP-binding C-terminal fragment, not the N-terminal fragment, lowered ubiquitination (Fig. 3F, lanes 4–5 versus 2). Similar results were obtained in *MLH1* proficient and deficient HeLa cells (Supplementary Fig. S4E). These data demonstrate that MLH1 directly binds YAP, stabilizing it by inhibiting both its phosphorylation and subsequent ubiquitin-mediated degradation.

### MLH1 binding facilitates YAP nuclear localization

**Figure 4.**
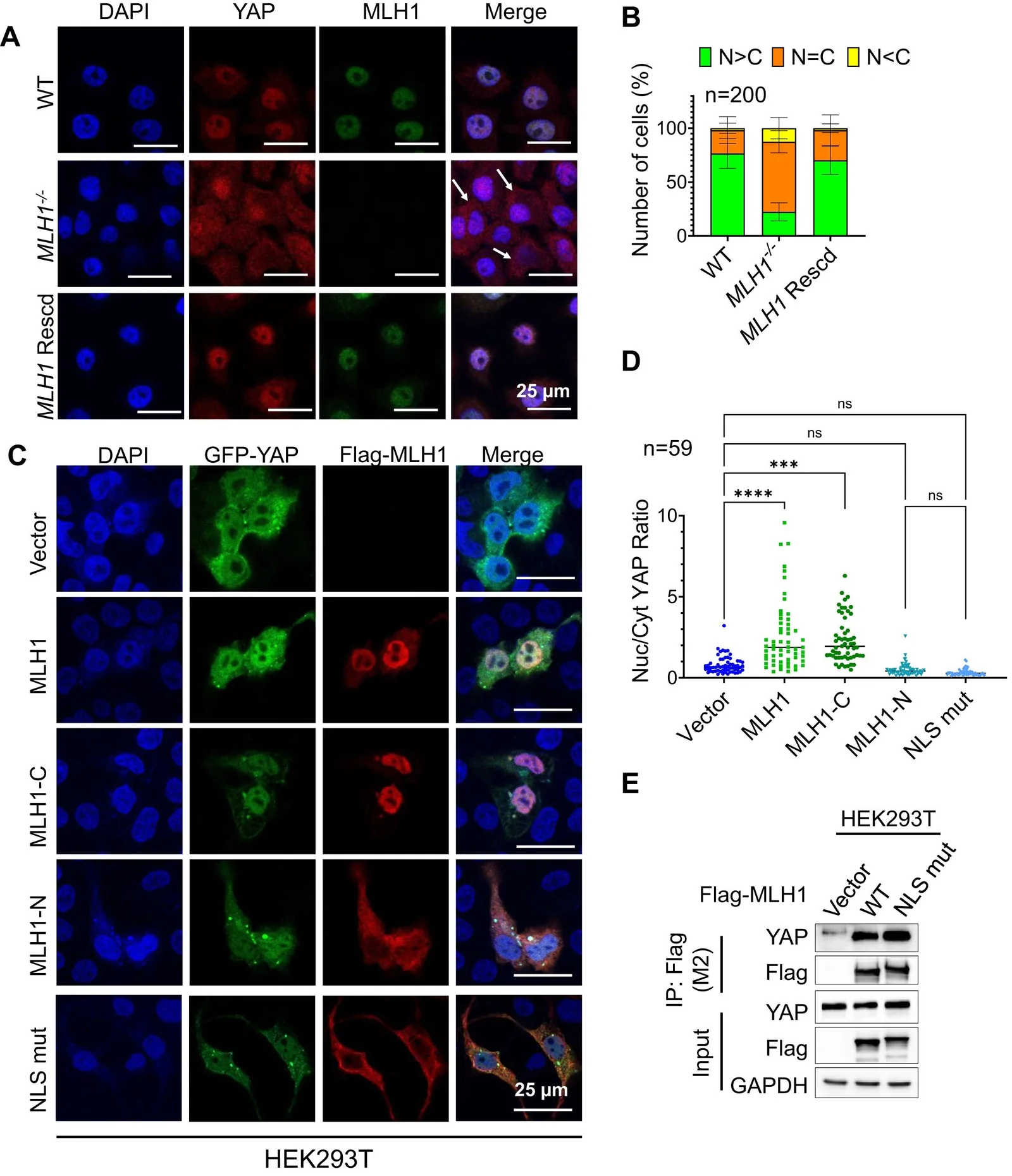
The MLH1–YAP interaction promotes YAP nuclear localization. (**A**) Representative immunofluorescence confocal images showing impaired nuclear localization of endogenous YAP in *MLH1^−/−^* HeLa cells compared with WT or *MLH1*-rescued cells. Nuclei are stained with DAPI. The white arrows point to representative cells with predominantly cytoplasmic YAP localization. Scale bar, 25 µm. (**B**) Quantification of YAP subcellular localization from the experiments in panel (A). Cells were categorized as N > C (nuclear signal > cytoplasmic), N = C (nuclear signal = cytoplasmic), or N < C (nuclear signal < cytoplasmic). (**C**) Confocal analysis showing that the nuclear translocation of ectopically expressed GFP-YAP is promoted by co-expression of full-length MLH1 (Flag-MLH1) or its C-terminal fragment (Flag-MLH1-C), but not by an empty vector, the N-terminal fragment (Flag-MLH1-N), or an NLS-mutated MLH1 (Flag-MLH1-NLS-mut). (**D**) Quantitative analysis of the nuclear-to-cytoplasmic (Nuc/Cyt) fluorescence intensity ratio of GFP-YAP from the experiment in panel (C) (*n* = 59 cells per condition). Data are presented as individual data points. Statistical significance was determined by one-way ANOVA. *, *P <* 0.05; **, *P <* 0.01; ***, *P <* 0.001; ^****^, *P <* 0.0001. (**E**) Co-IP-Western analysis confirming that the NLS mutation in MLH1 does not disrupt its interaction with YAP in HEK293T cell lysates.

Given that phosphorylation of YAP drives its association with 14-3-3 and subsequent cytoplasmic retention [26, 37, 38], we next investigated whether MLH1 regulates this interaction. Co-IP experiments showed that YAP-14-3-3 association was markedly increased in the absence of MLH1 (Supplementary Fig. S5A and B), indicating that MLH1 deficiency promotes YAP cytoplasmic sequestration. Consistent with this observation, immunofluorescence analysis in HeLa cells revealed predominantly nuclear YAP in WT and *MLH1*-rescued cells, whereas YAP was retained in cytoplasm in *MLH1^−/−^* cells (Fig. 4A and B). This pattern was confirmed in HCT116 and 293T models (Supplementary Fig. S5C and D), demonstrating that MLH1 is required for efficient YAP nuclear localization.

To determine whether this requires direct binding, we examined YAP’s subcellular localizations in *MLH1*-silenced 293T cells co-expressing GFP-YAP with full-length Flag-MLH1, Flag-MLH1-C (C-terminal fragment), or Flag-MLH1-N (N-terminal fragment). While YAP remained cytoplasmic in control cells (in the absence of MLH1), co-expression of full-length MLH1 or its YAP-binding C-terminal fragment (MLH1-C) restored robust nuclear localization, with clear nuclear co-localization (Fig. 4C and D). In contrast, the non-binding N-terminal fragment (MLH1-N) failed to rescue YAP nuclear accumulation (Fig. 4C and D). These results suggest that the MLH1–YAP interaction is essential for efficient YAP nuclear localization.

While MLH1 possesses defined nuclear localization signal (NLS) sequences, the requirement for a functional NLS in YAP is unclear. We next asked whether YAP nuclear import depends on MLH1’s NLS. We expressed an MLH1 NLS mutant (KRGPTSSNPRKRHR→ASGPTSSNPAAAHA) in 293T cells. This mutant retained the ability to bind YAP and inhibit its phosphorylation similarly to WT MLH1 (Fig. 4E and Supplementary Fig. S5E). However, the mutant failed to promote YAP nuclear import, with YAP predominantly detected in the cytoplasm (Fig. 4C and D). These results indicate that YAP nuclear translocation depends on both its physical interaction with MLH1 and a functional MLH1 NLS.

To further determine whether MLH1-driven YAP nuclear localization strictly requires suppression of LATS1-mediated YAP phosphorylation, we introduced the phosphorylation-deficient mutant GFP-YAP-S127A in WT, *MLH1*^−/−^, and *MLH1*-rescued HeLa cells. Strikingly, MLH1 ablation markedly diminished the nuclear enrichment of YAP-S127A, whereas restoration of MLH1 expression fully restored this nuclear phenotype (Supplementary Fig. S5F and G). These findings indicate that although inhibiting YAP phosphorylation, which stabilizes the protein, is a prerequisite for its nuclear accumulation, this process remains insufficient in the absence of MLH1, underscoring an essential, phosphorylation-independent role of MLH1 in facilitating YAP nuclear localization.

### MLH1 promotes YAP–TEAD transcriptional activity

**Figure 5.**
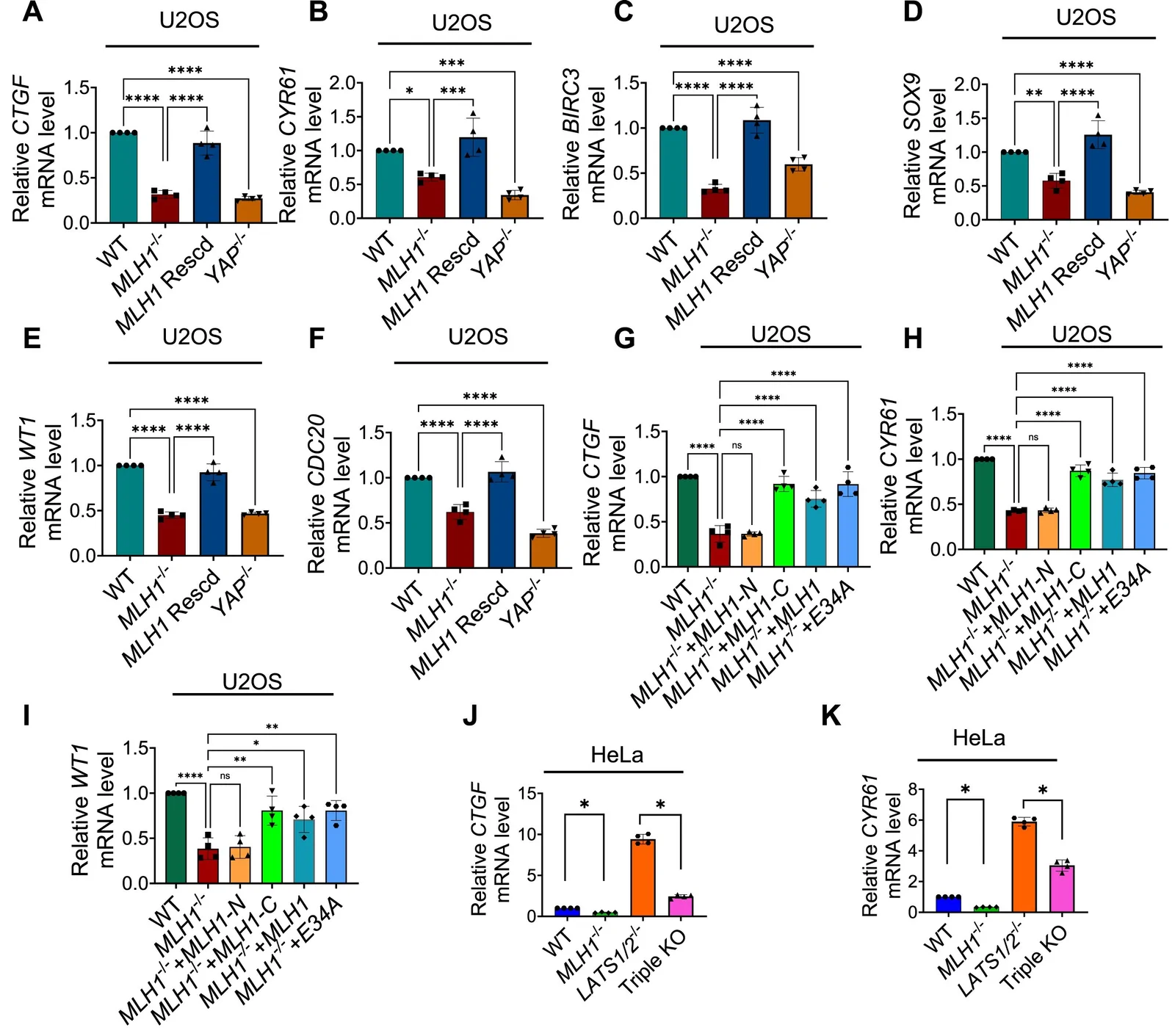
MLH1 promotes YAP transcriptional coactivator activity. qRT-PCR analyses showing mRNA levels of YAP target genes *CTGF* (**A**), *CYR61* (**B**), *BIRC3* (**C**), *SOX9* (**D**), *WT1* (**E**), and *CDC20* (**F**) in WT, *MLH1^−/−^, MLH1-*rescued *MLH1^−/−^*, and *YAP^−/−^* U2OS cells, as indicated. qRT-PCR analyses showing mRNA levels of *CTGF* (**G**), *CYR61* (**H**), and *WT1* (**I**) in WT, *MLH1^−/−^*, and *MLH1^−/−^* transfected with Flag-MLH1 N-terminal (N), C-terminal (C), MLH1, or MLH1-E34A U2OS cells. qRT-PCR analyses showing mRNA levels of YAP target genes *CTGF* (**J**) and *CYR61* (**K**) in WT, *MLH1^−/−^, LATS1/2^−/−^*, and *MLH1/LATS1/LATS2^−/−^* (triple KO) HeLa. The above data are from four biological replicates. Each biological replicate contained three technical replicates. Data points represent the mean of technical replicates; error bars denote SD. Statistical significance in panels (A–I) was determined by one-way ANOVA. Statistical significance between WT and *MLH1^−/−^, LATS1/2^−/−^*, and triple KO in panels (J) and (K) was determined by a two-tailed unpaired *t*-test. *, *P <* 0.05; **, *P <* 0.01; ***, *P <* 0.001; ^****^, *P <* 0.0001.

Given that MLH1 deficiency impairs YAP stability and nuclear localization, we hypothesized that MLH1 is required for YAP-dependent transcription. Analysis of mouse testes revealed significantly reduced mRNA levels of key YAP-target genes critical for testis development, including *Wt1, Sox9, Cdc20, Bmp4, Ctgf*, and *Cyr61* [27, 39–46] in *Mlh1^−/−^* versus WT animals, while *Yap* mRNA was unchanged (Supplementary Fig. S6A–G). Similarly, in U2OS cells, mRNA levels of YAP-regulated genes (*CTGF, CYR61, BIRC3, SOX9, WT1*, and *CDC20*) were significantly lower in *MLH1^−/−^* cells compared with WT controls and were rescued by MLH1 restoration (Fig. 5A–F), a phenotype mirrored *YAP^-/-^* cells (Fig. 5A–F and Supplementary Fig. S6H–K). This effect was consistent across TM4 (Supplementary Fig. S6H–K) and HCT116 models (Supplementary Fig. S6L–N). Critically, non-YAP targets (*RPLP0, RPL13A, ACTB*) were unaffected by *MLH1* depletion (Supplementary Fig. S6O–Q). We conclude that MLH1 specifically enables YAP’s transcriptional co-activator function.

To determine whether this regulation depends on MLH1’s canonical MMR function, we used an ATPase-defective MLH1-E34A mutant, which is MMR-deficient [47–49]. This mutant retained WT binding to YAP (Supplementary Fig. S6R) and fully restored expression of YAP target genes *CTGF, CYR61*, and *WT1* in *MLH1*^−/−^ U2OS cells (Fig. 5G–I), comparable to WT MLH1. The YAP-binding C-terminal fragment (MLH1-C) similarly rescued transcription, whereas the non-interacting N-terminal fragment (MLH1-N) failed (Fig. 5G–I). Both WT and E34A MLH1 also reduced YAP phosphorylation and restored CYR61 protein levels in *MLH1*^−/−^ HeLa cells (Supplementary Fig. S6S). These data suggest that MLH1 regulates YAP’s co-transcriptional activity via direct protein–protein interaction, independent of its canonical MMR function.

We next asked whether MLH1 regulates YAP function independently of LATS1/2. To address this, we generated *LATS1/2*^−/−^ and *MLH1/LATS1/LATS2*^−/−^ (triple knockout) HeLa cells. As expected, deletion of LATS1/2 completely abolished YAP phosphorylation in both WT and *MLH1*^−/−^ cells (Supplementary Fig. S6T). However, loss of *MLH1* continued to reduce YAP protein stability and impair YAP nuclear localization even in the complete absence of LATS1/2-mediated YAP phosphorylation (Supplementary Fig. S6U–X). Consistent with these observations, expression of the canonical YAP target genes CTGF and CYR61 remained significantly lower in triple-knockout cells than in *LATS1/2*^−/−^ cells (Fig. 5J and K). Together, these findings demonstrate that MLH1 regulates YAP stability, nuclear localization, and transcriptional activity through mechanisms that extend beyond the suppression of LATS1/2-mediated phosphorylation.

**Figure 6.**
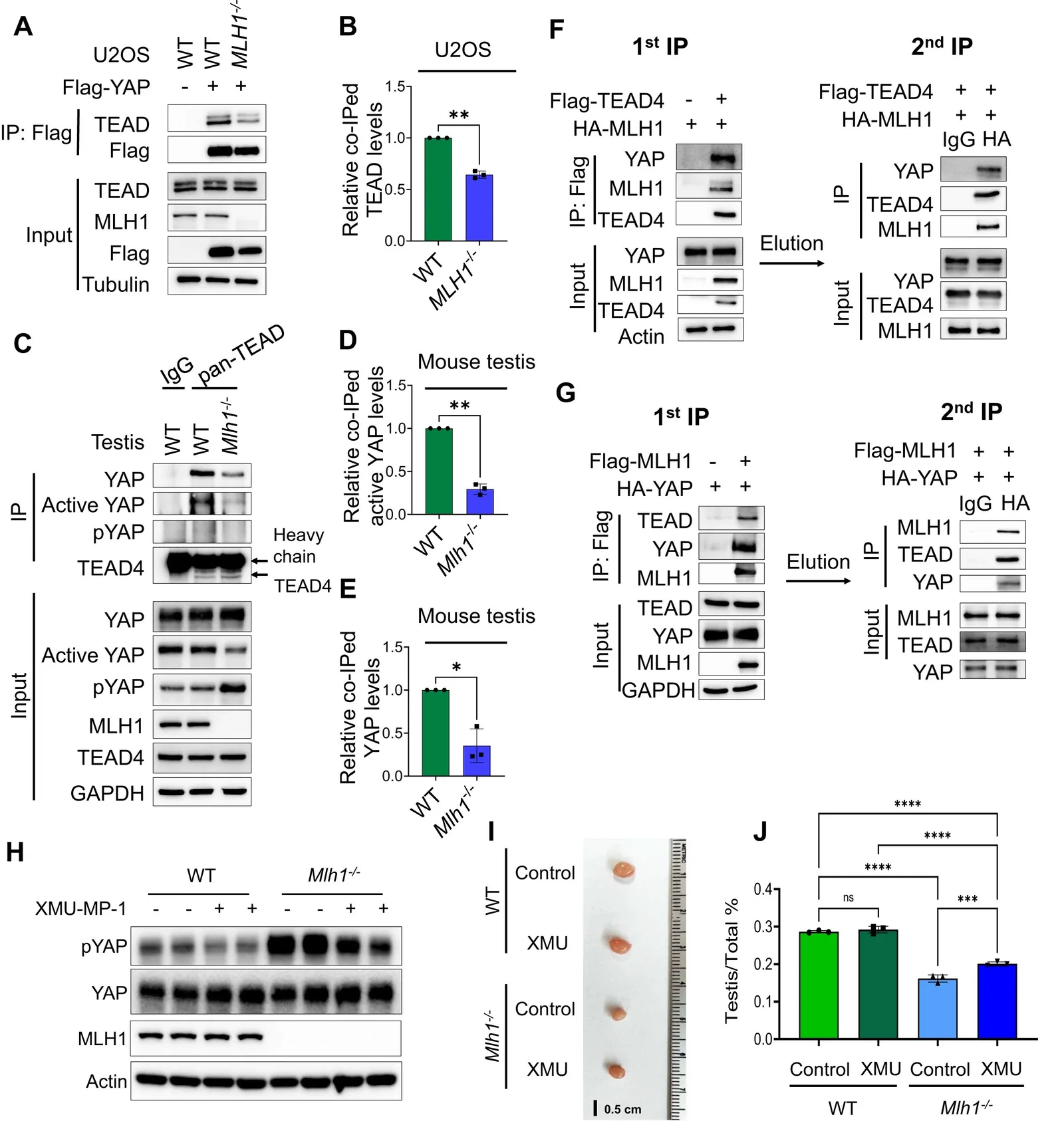
YAP, MLH1, and TEAD4 form a ternary complex. (**A**) Co-IP Western analysis showing the interaction between transfected Flag-YAP and endogenous TEAD in WT and *MLH1^−/−^* U2OS cells. Lysates were prepared 48 h post-transfection and immunoprecipitated with anti-Flag beads. (**B**) Quantification of co-IPed TEAD levels from three independent experiments as shown in panel (A). (**C**) Co-IP of endogenous proteins from 2-week-old mouse testis lysates using an anti-pan-TEAD antibody reveals reduced association of TEAD4 with both active (non-phosphorylated) and total YAP in *Mlh1*^−/−^ versus WT testes. Quantification of relative co-IPed active YAP (**D**) or total YAP (**E**) levels in panel (C) (*n* = 3 biological replicates). (F, G) Sequential two-step co-IP Western analysis demonstrating the formation of YAP–MLH1–TEAD4 ternary complex. HA-MLH1 and Flag-TEAD4 (**F**) or HA-YAP and Flag-MLH1 (**G**) were co-expressed in HEK293T cells. Lysates were subjected to a first IP with anti-Flag beads; the eluates were then used for a second IP with anti-HA antibody or control IgG. (**H**) Western blot analysis of YAP phosphorylation status in testis lysates from WT and *Mlh1*^−/−^ mice treated daily via I.P. injection with 1 mg/kg XMU-MP-1 or vehicle for 20 days, starting at postnatal day 18. (**I**) Gross morphology of testes from WT and *Mlh1*^−/−^ mice with or without XMU-MP-1 treatment. Scale bar, 0.5 cm. (**J**) Quantification of testis weight from panel (I). Data are presented as individual data points; significance was determined by two-tailed unpaired *t*-test with Welch’s correction (B, D, E) or one-way ANOVA (**J**); *, *P* < 0.05; **, *P* < 0.01; ***, *P* < 0.001; ^****^, *P* < 0.0001.

YAP exerts its transcriptional function by binding TEAD transcription factors [27]. To determine whether MLH1 facilitates YAP’s transcriptional activity by enhancing YAP–TEAD binding, we performed co-IP assay. The results showed that the YAP–TEAD interaction was significantly weakened in *MLH1*^−/-^ cells compared with WT controls (Fig. 6A and B). This finding was conserved across species, as a similarly reduced interaction was detected in *Mlh1*^−/−^ mouse testes (Fig. 6C–E). Our Co-IP results also revealed that this interaction was specific to non-phosphorylated (active) YAP (Fig. 6C and D and Supplementary Fig. S7A and B) and was rescued by MLH1 re-expression (Supplementary Fig. S7C and D). Reciprocal co-IP experiments revealed that MLH1, YAP, and TEAD4 form a ternary complex (Fig. 6F and G). Together, these findings establish that MLH1 directly facilitates YAP–TEAD complex formation and transcriptional activity, independent of its role in MMR.

### MST1/2 inhibitor partially restores impaired testis development in Mlh1−/− mice

To further prove that YAP inactivation underlies testis developmental defect in *Mlh1*^−/−^ mice, we inhibited the upstream Hippo pathway kinases MST1/2. *Mlh1*^−/−^ and WT mice were treated daily with the MST1/2 inhibitor XMU-MP-1 from postnatal day 18 for 20 days. This treatment significantly reduced YAP phosphorylation in *Mlh1*^−/−^ testes (Fig. 6H) and led to a partial but significant rescue of testis size compared with untreated knockout controls (Fig. 6I and J). However, the rescued testis size in treated *Mlh1*^−/−^ mice remained smaller than in WT controls (Fig. 6I and J). This incomplete rescue is likely attributable to two factors: first, the persistent impairment of YAP nuclear localization and transcriptional co-activator function in the absence of MLH1, which cannot be fully compensated for by limiting YAP phosphorylation alone; and second, the established cell-autonomous meiotic defect in *Mlh1*^−/−^ spermatocytes, which independently reduces testis cellularity by the experimental endpoint. These results demonstrate that pharmacological inhibition of the Hippo pathway partially restores YAP function and ameliorates testis growth defects in *Mlh1*^−/−^ mice, establishing YAP dysregulation as a key contributor to the developmental phenotype.

## Discussion

MLH1 deficiency causes testis hypoplasia and male infertility in mice [12, 13]. However, its involvement in organ development has remained unknown. This study elucidates the mechanism underlying testis hypoplasia in *Mlh1*-deficient mice, revealing a critical and previously unrecognized role for MLH1 in organ development through regulation of the Hippo-YAP signaling pathway. We demonstrate that MLH1 promotes YAP activity via direct physical interaction, operating independently of its canonical DNA MMR function [12, 13].

**Figure 7.**
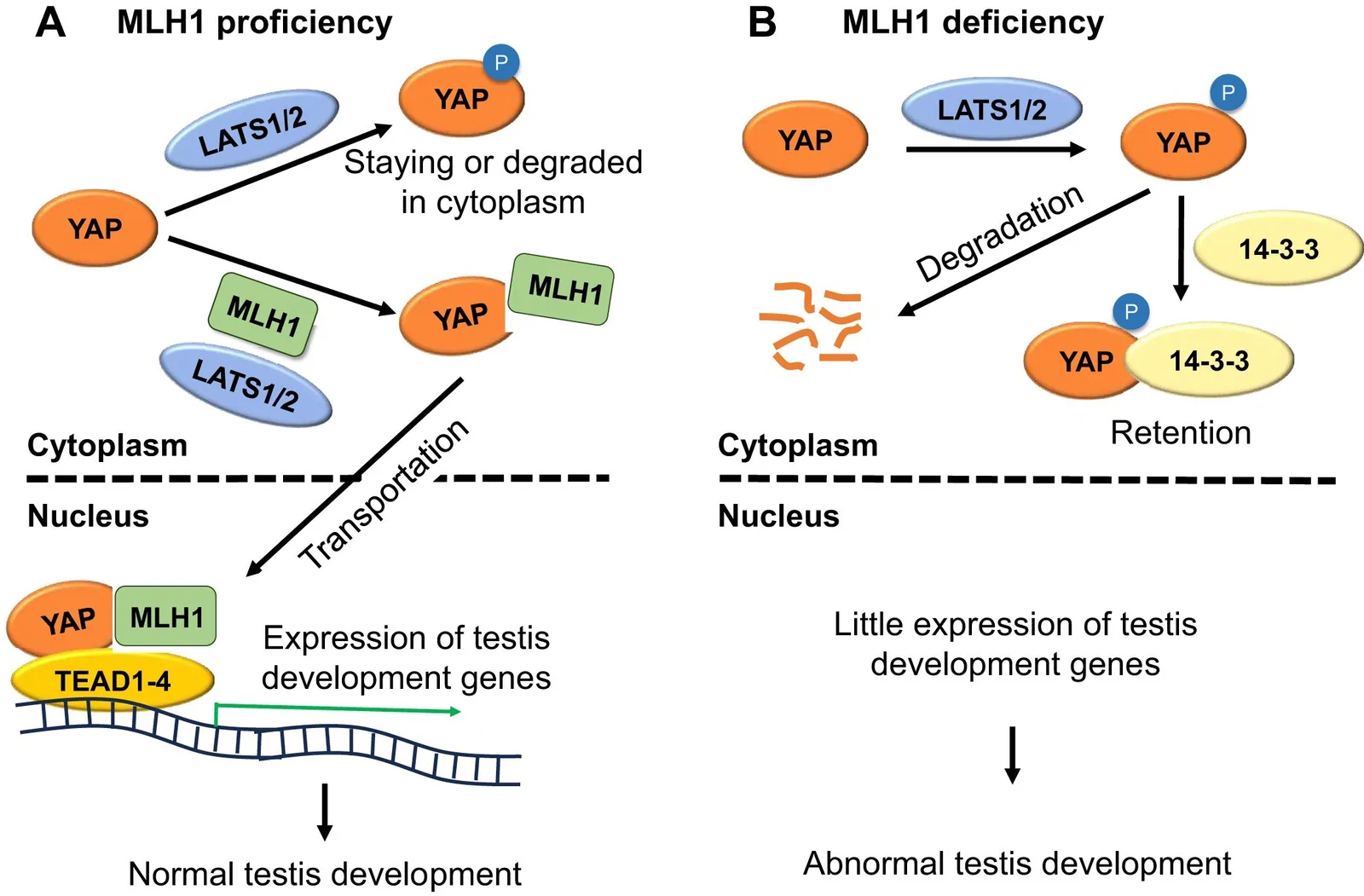
Proposed mechanism for male infertility caused by MLH1 deficiency. (**A**) Under normal conditions, YAP interacts with both LATS1 and MLH1. The MLH1–YAP interaction inhibits LATS1-mediated phosphorylation of YAP, promoting its nuclear translocation. Inside the nucleus, YAP forms a ternary complex with TEAD and MLH1 to activate the transcription of genes essential for testis development. (**B**) In the absence of MLH1, the inhibitory effect on LATS1 is lost. Unopposed LATS1 phosphorylates YAP, leading to either ubiquitin-mediated proteasomal degradation or cytoplasmic retention through 14-3-3 binding. This prevents YAP nuclear localization and the formation of the transcriptional complex, abolishing expression of critical testis development genes and resulting in testicular hypoplasia.

Our data support a model summarized in Fig. 7, which operates through three coordinated functions. First, MLH1 binds to the WW domains of YAP in the cytoplasm, directly competing with the upstream kinase LATS1 to inhibit YAP phosphorylation [25, 36] (Fig. 7A). Reduced phosphorylation limits YAP association with 14-3-3, thereby preventing its cytoplasmic retention and degradation [26]. Second, MLH1 acts as a nuclear shuttle; utilizing its own NLS, it facilitates YAP nuclear import, a role supported by similar findings for other transport receptors such as IPO7 [50]. Importantly, YAP-S127A remained dependent on MLH1 for efficient nuclear localization, and MLH1 loss continued to impair YAP stability, nuclear localization, and transcriptional activity even in LATS1/2-deficient cells, indicating that MLH1 regulates YAP through mechanisms extending beyond inhibition of LATS1-mediated phosphorylation. Third, within the nucleus, MLH1 integrates into the transcriptional machinery by forming a stable ternary complex with YAP and TEAD4. This complex is essential for activating target genes (*CTGF, CYR61, SOX9*, and *WT1*) crucial for testis development. Conversely, in the absence of MLH1 (Fig. 7B), YAP is constitutively phosphorylated by LATS1, leading to cytoplasmic retention via 14-3-3 binding, ubiquitin-mediated proteasomal degradation, and failure to activate the transcriptional program required for normal testicular growth.

Notably, the endogenous MLH1–YAP interaction decreased from 2- to 8-week-old mouse testes (Fig. 2E and F), suggesting that MLH1-mediated regulation of YAP is particularly important during early postnatal testicular development, when proliferation and differentiation are more active. However, these Co-IP analyses were performed using whole-testis lysates; we cannot exclude the possibility that age-dependent changes in cellular composition also contribute to the reduced interaction. During postnatal maturation, differentiated spermatids and spermatozoa become increasingly abundant, whereas proliferative Sertoli cells and early germ cells account for a smaller proportion of the tissue. Nonetheless, these findings support a model in which MLH1–YAP signaling plays its most prominent role during early postnatal testicular development.

Our findings delineate two distinct pathogenic axes in MLH1 deficiency: (i) the newly identified somatic pathway, in which disrupted MLH1–YAP signaling impairs testicular organogenesis, and (ii) the well-established germ cell-autonomous pathway, in which loss of MLH1’s meiotic function causes infertility [12, 13]. The specificity of the developmental defect for the testis is likely explained by the uniquely high co-expression of MLH1 and YAP in this tissue (and the liver) (Supplementary Fig. S1G). Although we observed no liver phenotype, YAP target genes in this organ are implicated in processes like fibrosis [51], regeneration [52], and tumor suppression [53], suggesting tissue-specific transcriptional programs. How MLH1-deficiency influences these processes in the liver remains to be investigated.

## Supplementary Material

gkag883_Supplemental_Files

## Data Availability

All data are available in the main text and/or Supplementary Materials.
