# Supplementary material for: Mismatch repair protein MLH1 controls testis development by regulating the Hippo-YAP signaling pathway
Source: Nucleic Acids Res. 2026 Sep 9;54(17):gkag883. doi: 10.1093/nar/gkag883 (PMC13554284; doi:10.1093/nar/gkag883)
Supplement: Supplementary file 1 [file gkag883_supplemental_files.zip › Revised Suppl Figures for NAR-01035-Q-2026.R1.pdf]

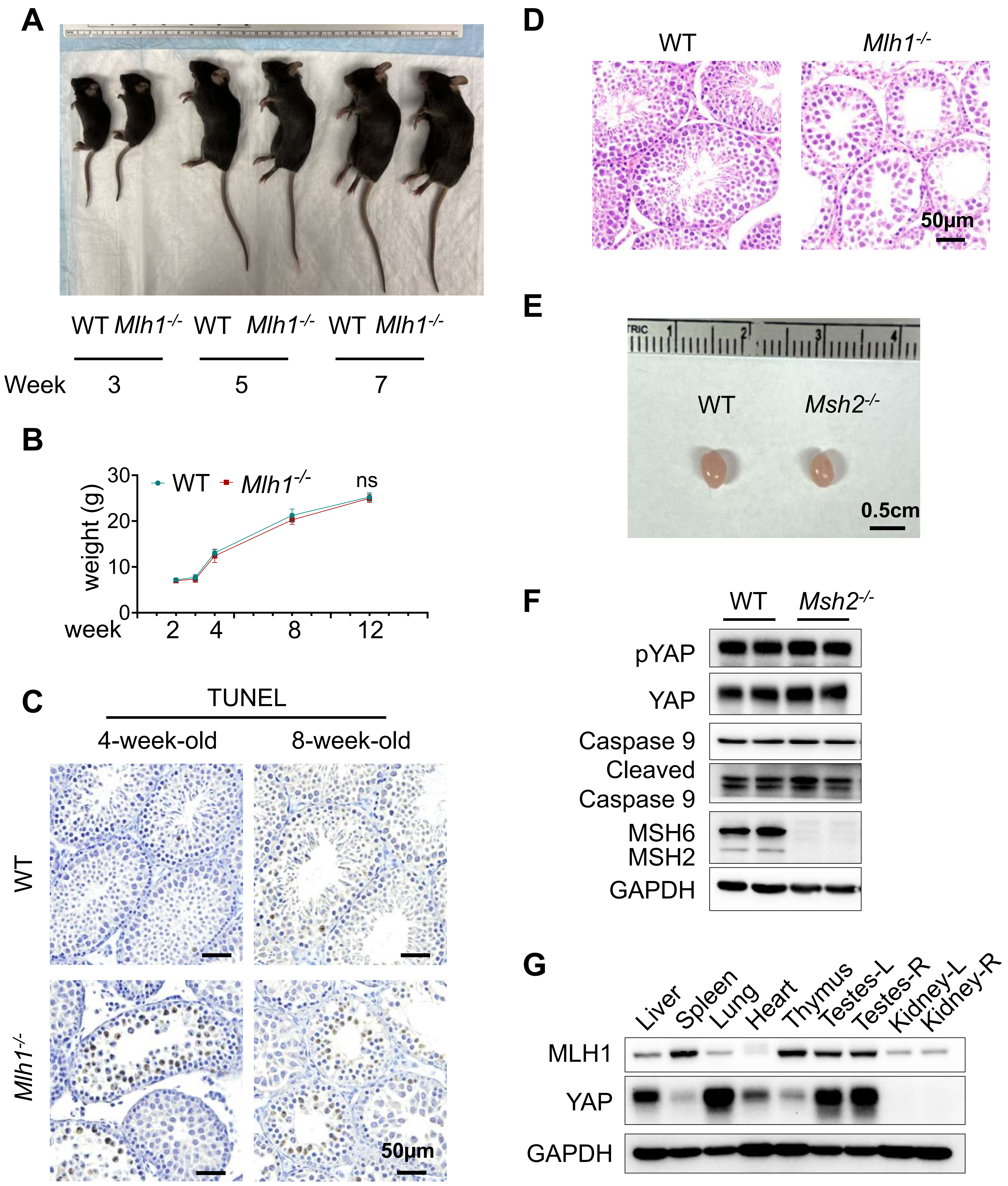

Fig. S1. *Mlh1*$^{-/-}$ mice exhibit smaller testes and activated Hippo-YAP pathway. (A) Body size of wild type (WT) and *Mlh1*$^{-/-}$ male mice at week 3, 5 or 7, as indicated. (B) Growing curve of body weight for both WT and *Mlh1*$^{-/-}$ mice. Five WT mice and five *Mlh1*$^{-/-}$ mice were used at each time point. The *P* value was determined by two-tailed unpaired *t* test with Welch's correction. (C) TUNEL assay showing apoptosis in the testis of *Mlh1*$^{-/-}$ mice at weeks 4 and 8, as indicated. (D) Hematoxylin and eosin (H&E) staining of WT and *Mlh1*$^{-/-}$ testis at week 6. (E) Gross morphology of WT and *Msh2*$^{-/-}$ testes at week 4. The scale bar is 0.5 cm. (F) Western blots showing Hippo-YAP signaling pathway in WT and *Msh2*$^{-/-}$ testes at week 4. (G) Western blotting analysis showing expression of MLH1 and YAP in the indicated mouse tissues. L indicates left side, and R indicates right side.

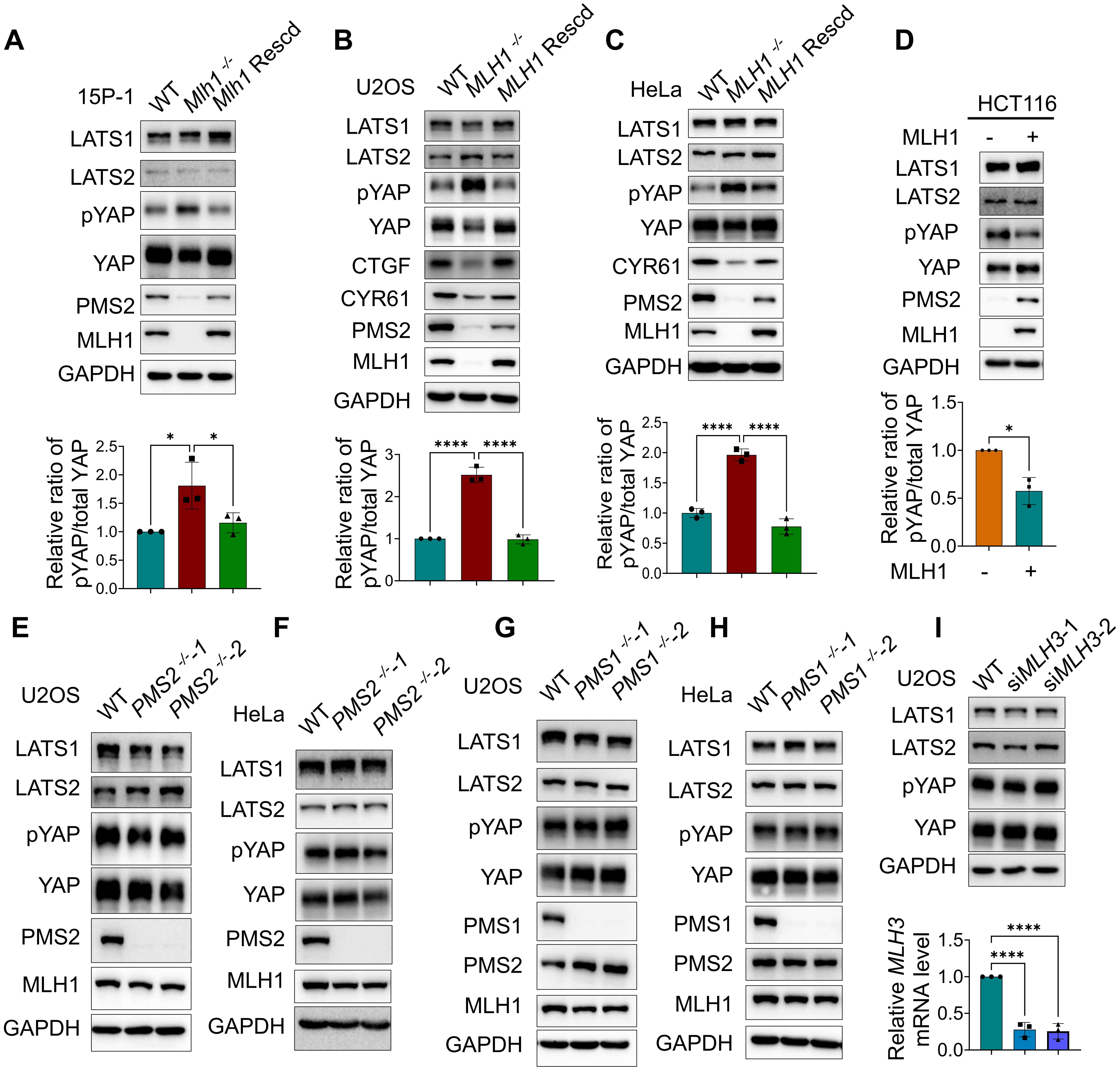

Fig. S2. Increased YAP phosphorylation in *MLH1* deficient cells. (A) Western blots showing increased phosphorylation of YAP (pYAP) in *Mlh1*$^{-/-}$, as compared with WT and *Mlh1*-rescued (Rescd) *Mlh1*$^{-/-}$ 15P-1 cells. Percentage of pYAP in 15P-1 cells was calculated with three biological replicates. (B) Western blots showing increased phosphorylation of YAP (pYAP) and decreased expression of total YAP and YAP target genes (CTGF and CYR61) in *MLH1*$^{-/-}$ U2OS cells, as compared with WT and *MLH1*-rescued (Rescd) *MLH1*$^{-/-}$ U2OS cells. Percentage of pYAP in U2OS cells was calculated with three biological replicates. (C) Western blots showing increased phosphorylation of YAP (pYAP) and decreased YAP target gene (CYR61) in *MLH1*$^{-/-}$ HeLa cells, as compared with WT and *MLH1*-rescued (Rescd) *MLH1*$^{-/-}$ HeLa cells. Percentage of pYAP in HeLa cells was calculated with three biological replicates. (D) Western blots showing reduced pYAP in *MLH1*-rescued HCT116 cells in comparison with parental *MLH1*-deficient HCT116 cells. Percentage of pYAP in parental and *MLH1*-rescued HCT116 cells was calculated with three biological replicates. (E-I) Western blots comparing pYAP levels between WT and *PMS2*$^{-/-}$ (E, F), WT and *PMS1*$^{-/-}$ (G, H), or WT and *MLH3*-knockdown (I) U2OS or HeLa cells, as indicated. Expression levels of Hippo-YAP proteins LATS1 and LATS2, related MMR proteins are also shown. (I) qRT-PCR analyses showing *MLH3* mRNA levels in WT and *MLH3*-siRNA treated U2OS cells. The *P* value in (D) was determined by two-tailed unpaired *t* test with Welch's correction and the others were determined by one-way ANOVA test. A *P*-value below 0.05 is considered statistically significant (*$p < 0.05$, **$p < 0.01$, ***$p < 0.001$, ****$p < 0.0001$). Data are presented as individual values.

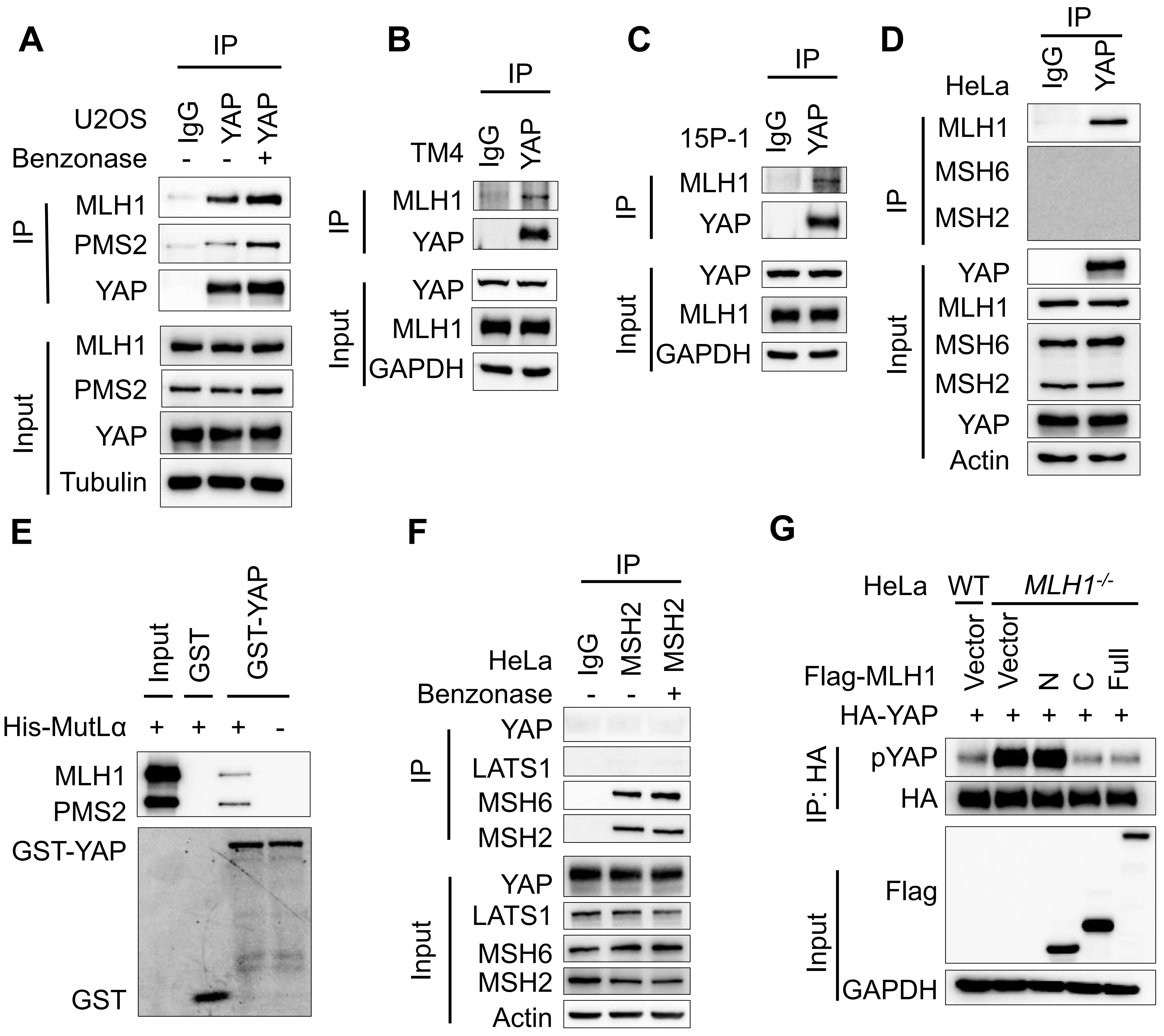

Fig. S3. MLH1 physically interacts with YAP. (A-D) Co-IP assays were performed in cell lysates derived from the indicated cell lines and antibodies, followed by Western blot analysis. (A) YAP interacts with MutLα in U2OS cells. (B) YAP interacts with MLH1 in TM4 cells. (C) YAP interacts with MLH1 in 15P-1 cells. (D) YAP interacts with MLH1, but not with other MMR proteins such as MSH2 or MSH6 in HeLa cells. (E) GST-pulldown assay showing interaction between purified YAP and MutLα. (F) Co-IP Western analysis showing no interaction between MSH2 and YAP. (G) Co-IP Western analysis showing inhibition of YAP phosphorylation by exogenous full-length MLH1 or C-terminal MLH1 in HeLa cells. HA-YAP was co-expressed with indicated Flag-full-length (Full) MLH1, Flag-N-terminal (N) or Flag-C-terminal (C) fragment in *MLH1*$^{-/-}$ HeLa cells. Cell lysates were obtained 48 hours post co-transfection, followed by Co-IP with anti-HA antibody and Western blotting analysis.

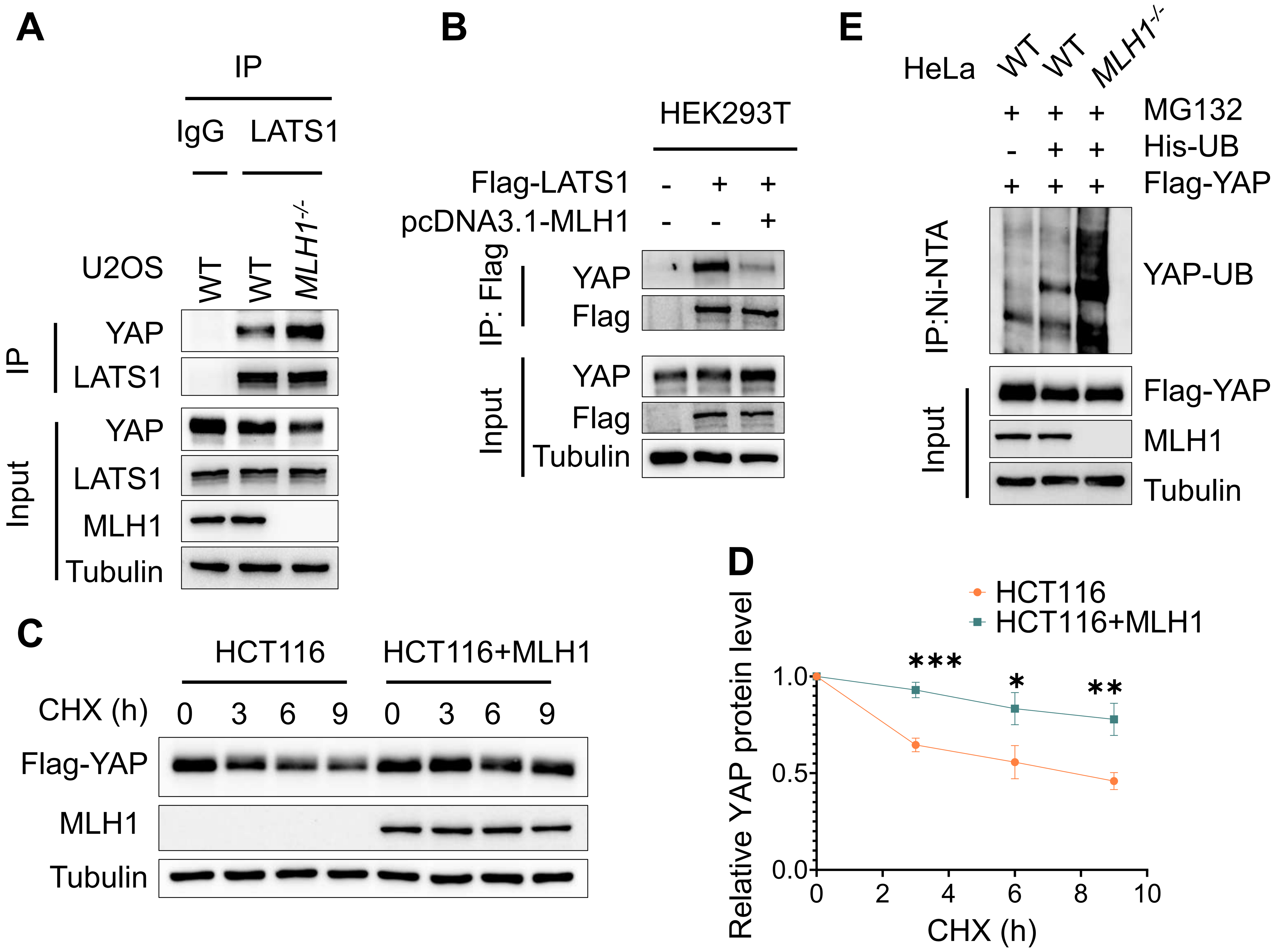

Fig. S4 The MLH1-YAP interaction elongates YAP's half-life. (A) Co-IP Western analysis showing enhanced binding between endogenous YAP and LATS1 in *MLH1*$^{-/-}$ U2OS cells. Cell lysates were subjected to Co-IP using an anti-LATS1 antibody, and the precipitates were analyzed by Western blotting. (B) Co-IP Western analysis showing reduced interaction between YAP and LATS1 in MLH1-overexpressed HEK293T cells. HEK293T cells were transfected with the plasmid expressing Flag-LATS1 in the absence or presence of MLH1, and cell lysates were subjected to Co-IP using an anti-Flag M2 beads, and the precipitates were analyzed by Western blotting. (C) Western blots showing longer half-life of YAP in *MLH1*-rescued HCT116 cells, in comparison to that in HCT116 cells. Both HCT116 and *MLH1*-rescued HCT116 cells were transfected with the plasmid expressing Flag-YAP for 24 hours and treated with cycloheximide (CHX, 100 μg/mL) for the indicated times. The level of Flag-YAP was detected by Western blotting. (D) Relative YAP levels, as shown in (C), with three independent assays. The Flag-YAP level at time 0 was used as a reference. Data are shown as means ± SD. The *P* value at each time point was determined by two-tailed unpaired *t* test with Welch's correction. (E) *In vivo* ubiquitination assay showing enhanced ubiquitination of YAP in *MLH1*$^{-/-}$ HeLa cells. Flag-YAP and His-ubiquitin were co-expressed in WT and *MLH1*$^{-/-}$ HeLa cells. Cells were treated with MG132 (10 μM) for 12h after plasmid transfection. Cell lysates were collected under denaturing conditions and the ubiquitinated proteins were pulled down using Ni-NTA agarose beads. The ubiquitination of Flag-YAP was detected by Western Blotting using an anti-Flag antibody.

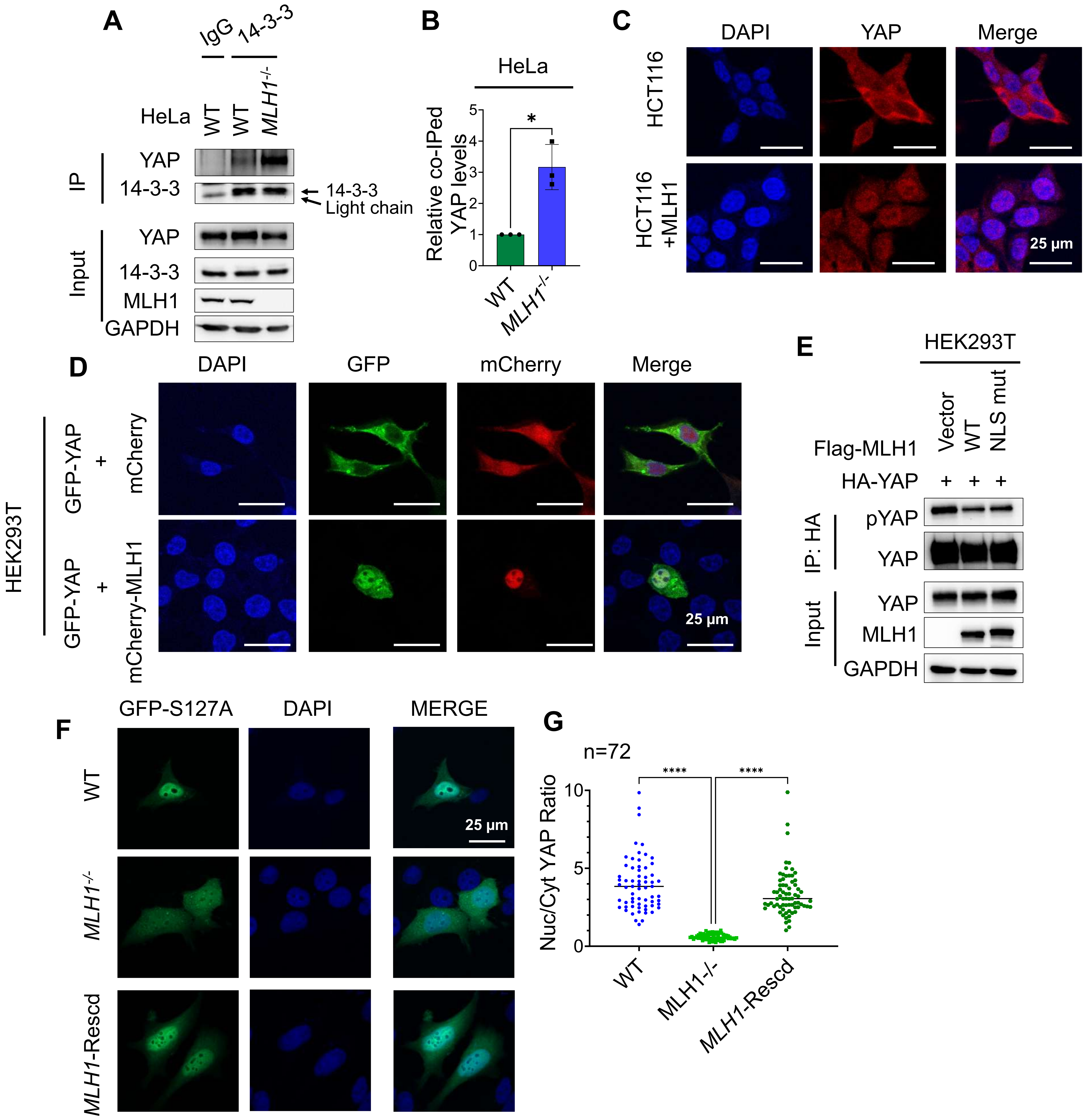

Fig. S5 The MLH1-YAP interaction facilitates YAP nuclear localization. (A) Co-IP Western analysis showing increased interaction between YAP and 14-3-3 in *MLH1*$^{-/-}$ HeLa cells. Cell lysates were subjected to Co-IP using an anti-pan-14-3-3 antibody, and the precipitates were analyzed by Western blotting. (B) Quantification of relative co-IPed YAP levels in (A). Data are from three biological replicates and are presented as individual data points. Statistical significance was determined by two-tailed unpaired *t*-test with Welch's correction. (C) Immunofluorescence confocal analysis showing increased nucleus localization of YAP in *MLH1*-rescued HCT116, in comparison with the parental HCT116. (D) Immunofluorescence confocal analysis showing increased nuclear localization of GFP-YAP in the presence of mCherry-MLH1 in HEK293T cells, as compared with mCherry vector-cells. (E) Co-IP Western analysis showing the pYAP levels in HEK293T cells expressing WT or NLS-mutated MLH1, as indicated. 293T cells were co-transfected with plasmid expressing HA-YAP and plasmid expressing Flag-WT MLH1 or NLS-mutated Flag-MLH1. Thirty-six hours post transfection, cell lysates were subjected to Co-IP with an anti-HA antibody, followed by Western blotting analysis. (F) Immunofluorescence confocal analysis showing impaired nuclear localization of GFP-YAP-S127A in *MLH1*$^{-/-}$ HeLa cells, as compared with WT and *MLH1*-rescued (Rescd) HeLa cells. Scale bar, 25 µm. (G) Quantitative analysis of the nuclear-to-cytoplasmic (Nuc/Cyt) fluorescence intensity ratio of GFP-YAP-S127A from the experiment in (F) (n=72 cells per condition). Data are presented as individual data points. Statistical significance was determined by one-way ANOVA. *, $p < 0.05$; **, $p < 0.01$; ***, $p < 0.001$; ****, $p < 0.0001$.

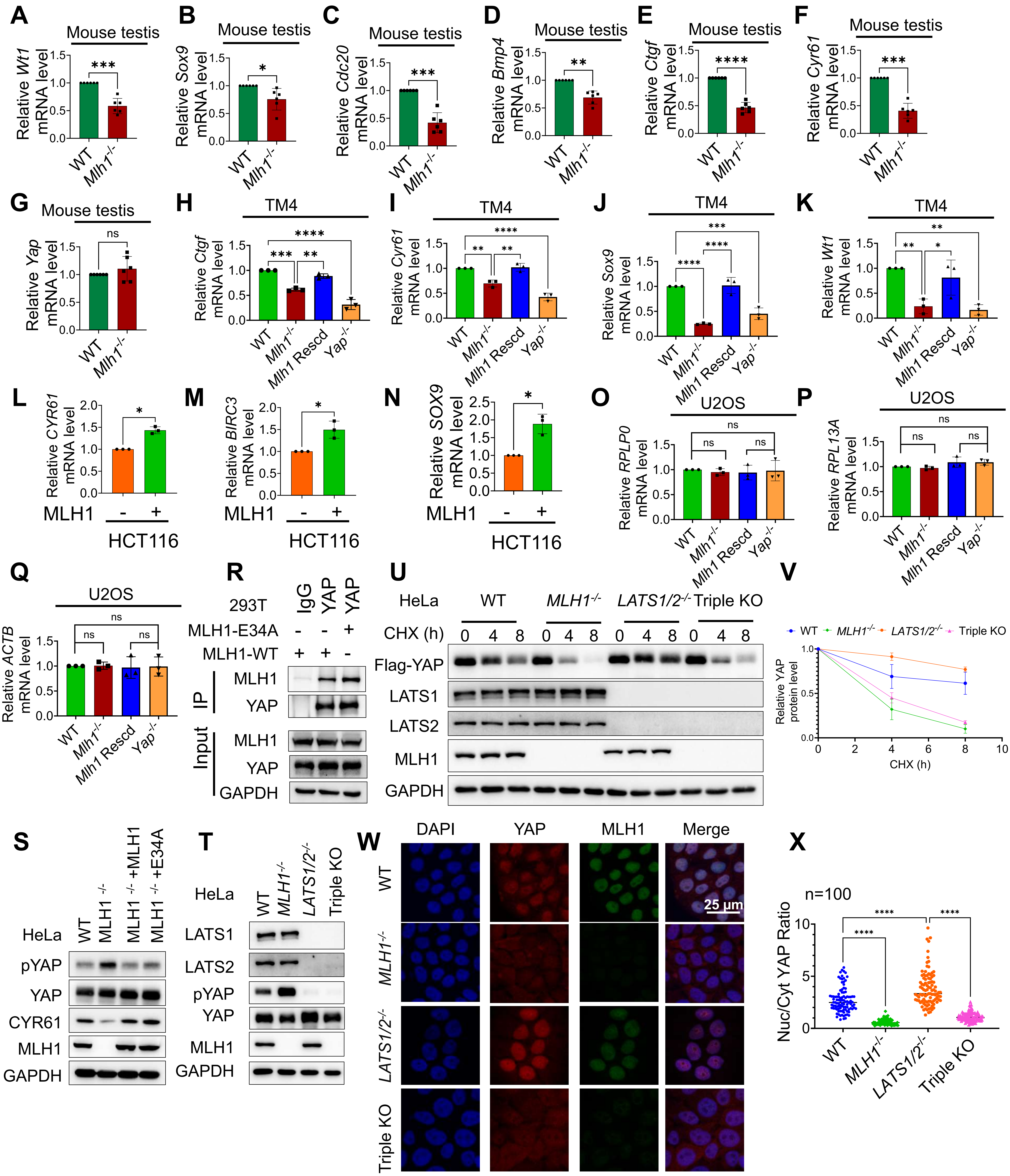

Fig. S6. MLH1 promotes YAP co-transcriptional activity. (A-K) qRT-PCR analyses showing mRNA levels of *Wt1* (A), *Sox9* (B), *Cdc20* (C), *Bmp4* (D), *Ctgf* (E), *Cyr61* (F) and *Yap* (G) in *Mlh1*$^{-/-}$ testis, and *Ctgf* (H), *Cyr61* (I), *Sox9* (J), *Wt1* (K) in WT, *Mlh1*$^{-/-}$, *Mlh1*-rescued *Mlh1*$^{-/-}$ and *Yap*$^{-/-}$ TM4 cells. (L-N) qRT-PCR analyses showing increased mRNA levels of *CYR61* (L), *BIRC3* (M) and *SOX9* (N) in *MLH1*-rescued (*MLH1*-rescd) HCT116 cells. (O-Q) qRT-PCR analyses showing unchanged mRNA levels of *RPLP0* (O), *RPL13A* (P), *ACTB* (Q) in WT, *MLH1*$^{-/-}$, *MLH1* rescued *MLH1*$^{-/-}$ and *YAP*$^{-/-}$ U2OS cells. For the above data, each biological replicate contains three technical repeats. Each individual data point represented the mean value of three technical replicates. (R) Co-IP Western analysis showing the interaction of YAP with MLH1-WT or MLH1-E34A in HEK293T cell lysates. (S) Western blots showing that increased pYAP and decreased expression of YAP target gene CYR61 in *MLH1*$^{-/-}$ HeLa cells were rescued by expression of either exogenous WT MLH1 or MLH1-E34A. (T) Western blot analysis confirming the deletion of LATS1, LATS2, and MLH1 in the indicated HeLa cell lines. YAP Ser127 phosphorylation was completely abolished in *LATS1/2*$^{-/-}$ and *MLH1/LATS1/LATS2*$^{-/-}$ (triple KO) cells. (U) Assessment of YAP protein stability. WT, *MLH1*$^{-/-}$, *LATS1/2*$^{-/-}$ and triple KO HeLa cells expressing Flag-YAP were treated with cycloheximide (CHX, 100 μg/mL) for the indicated times. Flag-YAP levels were analyzed by Western blot. (V) Quantification of residual Flag-YAP levels from three independent experiments shown in (U). Data are presented as mean ± SD. (W) Representative immunofluorescence confocal images showing endogenous YAP localization. Scale bar, 25 μm. (X) Quantitative analysis of the nuclear-to-cytoplasmic (Nuc/Cyt) fluorescence intensity ratio of YAP from the experiment in (W) (n=100 cells per condition). The *P* value was determined by either two-tailed unpaired *t* test with Welch's correction (A-G, L-N) or one-way ANOVA (H-K, O-Q, X). **p* < 0.05, ***p* < 0.01, ****p* < 0.001, *****p* < 0.0001.

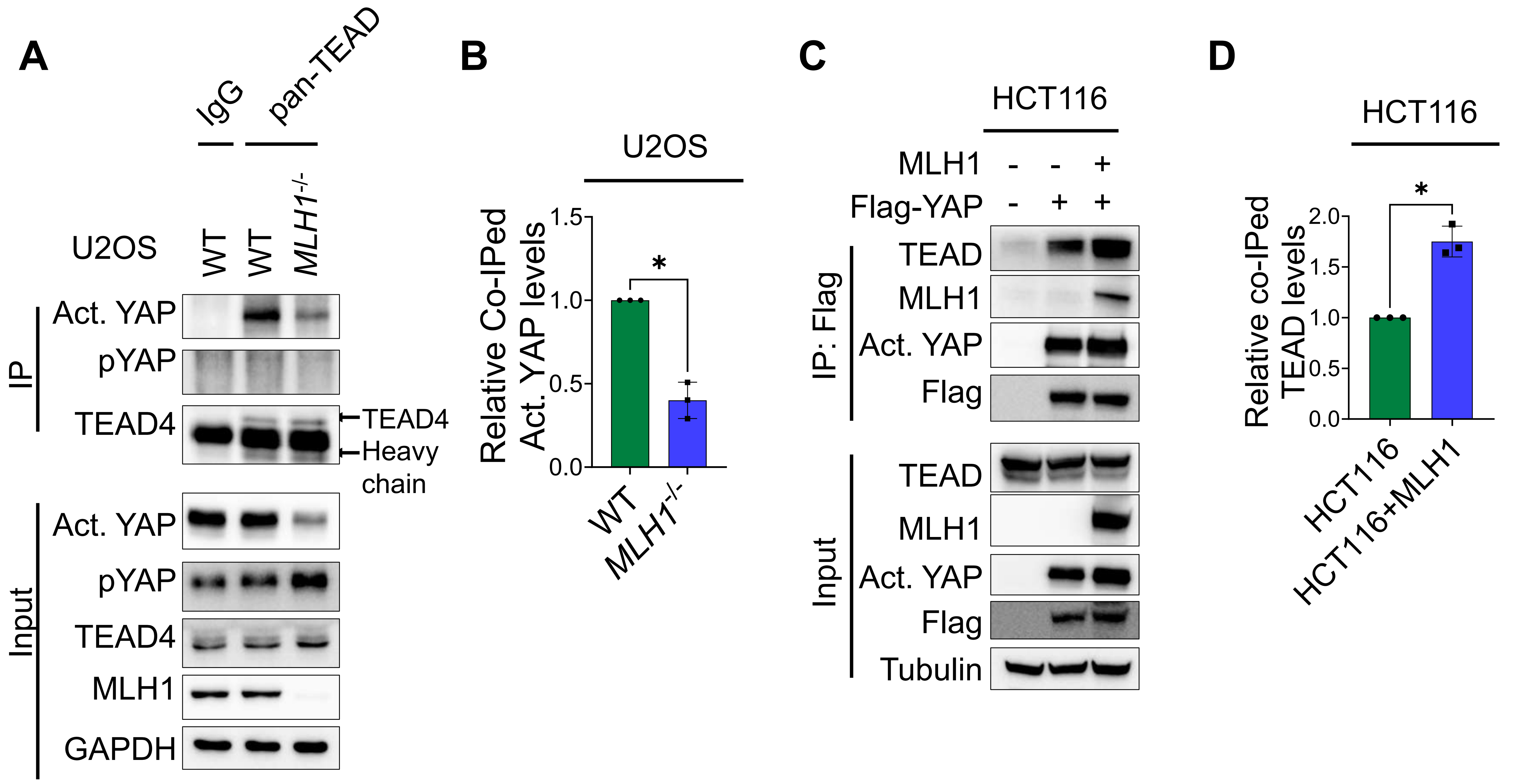

Fig. S7 YAP, MLH1 and TEAD4 form a ternary complex. (A) Co-IP Western analysis showing reduced interaction between active-YAP (Act. YAP) and TEAD in *MLH1*$^{-/-}$ U2OS cells. Cell lysates were subjected to Co-IP using an anti-pan-TEAD antibody, and the precipitates were analyzed by Western blotting. (Note: The molecular size of TEAD4 in human is slightly higher than that in mouse which is the reason the band for TEAD4 is above heavy chain in Fig. S7A but below heavy chain in Fig. 6C) (B) Quantification of relative co-IPed active YAP levels in (A). Data are from three biological replicates. (C) Co-IP Western analysis showing enhanced interaction between Flag-YAP and pan-TEAD in *MLH1*-rescued HCT116 cells. Cell lysates were subjected to Co-IP using anti-Flag M2 beads, and the precipitates were analyzed by Western blotting. (D) Quantification of relative co-IPed TEAD levels normalized to immunoprecipitated active YAP in (C). Data are from three biological replicates. Data in (B, D) were analyzed by a two-tailed unpaired *t*-test with Welch's correction. A *P*-value below 0.05 is considered statistically significant (*$p < 0.05$).
