# Supplementary material for: Mismatch repair protein MLH1 controls testis development by regulating the Hippo-YAP signaling pathway
Source: Nucleic Acids Res. 2026 Sep 9;54(17):gkag883. doi: 10.1093/nar/gkag883 (PMC13554284; doi:10.1093/nar/gkag883)
Supplement: Supplementary file 1 [file gkag883_supplemental_files.zip › Table S1.pdf]

Table S1. Reagents used in this study and their sources.

REAGENT or RESOURCES	SOURCES	IDENTIFIER
**Antibodies**		
Phospho-YAP (Ser127) Antibody	Cell Signaling Technology	Cat# 4911
YAP (D8H1X) Rabbit Monoclonal Antibody	Cell Signaling Technology	Cat# 14074
Pan-TEAD (D3F7L) Rabbit Monoclonal Antibody	Cell Signaling Technology	Cat# 13295
LATS1 (C66B5) Rabbit Monoclonal Antibody	Cell Signaling Technology	Cat# 3477
LATS2 (D83D6) Rabbit Monoclonal Antibody	Cell Signaling Technology	Cat# 5888
Caspase-9 (C9) Mouse Monoclonal Antibody	Cell Signaling Technology	Cat# 9508
GAPDH (D16H11) Rabbit Monoclonal Antibody	Cell Signaling Technology	Cat# 5174
CYR61 (E5W3H) Rabbit Monoclonal Antibody	Cell Signaling Technology	Cat# 39382
Cyr61/CCN1 Antibody (A-10)	Santa Cruz Biotechnology	Cat# sc-374129
CTGF Antibody (E-5)	Santa Cruz Biotechnology	Cat# sc-365970
MSH2 Antibody (D-6)	Santa Cruz Biotechnology	Cat# sc-376384
Ubiquitin Antibody (P4D1)	Santa Cruz Biotechnology	Cat# sc-8017
HA-Tag Antibody (F-7)	Santa Cruz Biotechnology	Cat# sc-7392
beta Actin Antibody (C4)	Santa Cruz Biotechnology	Cat# sc-47778
alpha Tubulin Antibody (B-5-1-2)	Santa Cruz Biotechnology	Cat# sc-23948
pan 14-3-3 Antibody (H-8)	Santa Cruz Biotechnology	Cat# sc-1657
Anti-active YAP1 antibody	Abcam	Cat# ab205270
Anti-MLH1 antibody	Abcam	Cat# ab92312
Purified Mouse Anti-Human MLH-1	BD Pharmingen	Cat# 550838
Purified Mouse Anti-PMS2	BD Pharmingen	Cat# 556415
Purified Mouse Anti-MSH6	BD Pharmingen	Cat# 610919
TEAD4 Polyclonal antibody	Proteintech	Cat# 12418-1-AP
PMS1 Monoclonal antibody	Proteintech	Cat# 68413-1-Ig
LATS2-Specific Polyclonal antibody	Proteintech	Cat# 20276-1-AP
ANTI-FLAG® M2 antibody, Mouse monoclonal	Sigma	Cat# F1804

**Oligonucleotides**	**Sequence**	
Mouse *Sox9* forward	CACACGTCAAGCGACCCATGAA	N/A
Mouse *Sox9* reverse	TCTTCTCGCTCTCGTTCAGCAG	N/A
Mouse *Wt1* forward	GGTTTTCTCGCTCAGACCAGCT	N/A
Mouse *Wt1* reverse	ATGAGTCCTGGTGTGGGTCTTC	N/A
Mouse *Bmp4* forward	GCCGAGCCAACACTGTGAGGA	N/A
Mouse *Bmp4* reverse	GATGCTGCTGAGGTTGAAGAGG	N/A
Mouse *Cdc20* forward	TTCGTGTTCGAGAGCGATTTG	N/A
Mouse *Cdc20* reverse	ACCTTGGAACTAGATTTGCCAG	N/A
Mouse *Ctgf* forward	TGCGAAGCTGACCTGGAGGAAA	N/A
Mouse *Ctgf* reverse	CCGCAGAACTTAGCCCTGTATG	N/A
Mouse *Cyr61* forward	GTGAAGTGCGTCCTTGTGGACA	N/A
Mouse *Cyr61* reverse	CTTGACACTGGAGCATCCTGCA	N/A
Mouse *Yap* forward	TGAGATCCCTGATGATGTACCAC	N/A
Mouse *Yap* reverse	TGTTGTTGTCTGATCGTTGTGAT	N/A
Mouse *Gapdh* forward	AGGTCGGTGTGAACGGATTTG	N/A
Mouse *Gapdh* reverse	GGGGTCGTTGATGGCAACA	N/A
Human *CYR61* forward	GGAAAAGGCAGCTCACTGAAGC	N/A
Human *CYR61* reverse	GGAGATACCAGTTCCACAGGTC	N/A
Human *CTGF* forward	CTTGCGAAGCTGACCTGGAAGA	N/A
Human *CTGF* reverse	CCGTCGGTACATACTCCACAGA	N/A
Human *AREG* forward	GCACCTGGAAGCAGTAACATGC	N/A
Human *AREG* reverse	GGCAGCTATGGCTGCTAATGCA	N/A
Human *WT1* forward	CGAGAGCGATAACCACACAACG	N/A
Human *WT1* reverse	GTCTCAGATGCCGACCGTACAA	N/A
Human *CDC20* forward	CGGAAGACCTGCCGTTACATTC	N/A
Human *CDC20* reverse	CAGAGCTTGCACTCCACAGGTA	N/A
Human *BIRC3* forward	GCTTTTGCTGTGATGGTGGACTC	N/A
Human *BIRC3* reverse	CTTGACGGATGAACTCCTGTCC	N/A

Human *SOX9* forward	AGGAAGCTCGCGGACCAGTAC	N/A
Human *SOX9* reverse	GGTGGTCCTTCTTGTGCTGCAC	N/A
Human *GAPDH* forward	GTCTCCTCTGACTTCAACAGCG	N/A
Human *GAPDH* reverse	ACCACCCTGTTGCTGTAGCCAA	N/A
Human *RPLP0* forward	TGGTCATCCAGCAGGTGTTCGA	N/A
Human *RPLP0* reverse	ACAGACACTGGCAACATTGCGG	N/A
Human *RPL13A* forward	CTCAAGGTGTTTGACGGCATCC	N/A
Human *RPL13A* reverse	TACTTCCAGCCAACCTCGTGAG	N/A
Human *ACTB* forward	CACCATTGGCAATGAGCGGTTC	N/A
Human *ACTB* reverse	AGGTCTTTGCGGATGTCCACGT	N/A
Human *MLH3* forward	CTGACTGTCCAGAAGGTGTTGG	N/A
Human *MLH3* reverse	TGAGCACACTGGAATGGCAGCT	N/A
Mouse *Mlh1* (sgRNA-1 for mouse gene)	CAACCAGGGCACCCTGATCA	N/A
Mouse *Mlh1* (sgRNA-2 for mouse gene)	CTAATTCAGATCCAAGACAA	N/A
Human *MLH1* (sgRNA-1 for human gene)	TGATAGCATTAGCTGGCCGC	N/A
Human *MLH1* (sgRNA-2 for human gene)	AGTGGTGAACCGCATCGCGG	N/A
Mouse *Yap* (sgRNA for mouse gene)	CTGAGGCACGTTGGCCGTCT	N/A
Human *YAP* (sgRNA for human gene)	CAGGGCCCGCCGTCCGGACC	N/A
Human *PMS2* (sgRNA-1 for human gene)	AATGCCGTGGGTCTCAAAGA	N/A
Human *PMS2* (sgRNA-2 for human gene)	ACGAGATCGCTGCAACACTG	N/A
Human *PMS1* (sgRNA-1 for human gene)	AATGCCGTGGGTCTCAAAGA	N/A
Human *PMS1* (sgRNA-2 for human gene)	ACGAGATCGCTGCAACACTG	N/A
Human *LATS1* (sgRNA-1 for human gene)	GCAGCCATCTGCTCTCGTCG	N/A
Human *LATS1* (sgRNA-2 for human gene)	GTAACACTCCTTACTTGAGG	N/A
Human *LATS2* (sgRNA-1 for human gene)	TTTCCTGCCACGACTTATTC	N/A
Human *LATS2* (sgRNA-2 for human gene)	GCCATCCAAGTCTTCGGTTC	N/A
